# Hydrogels Based on Chitosan and Nanoparticles and Their Suitability for Dyes Adsorption from Aqueous Media: Assessment of the Last-Decade Progresses

**DOI:** 10.3390/gels10030211

**Published:** 2024-03-21

**Authors:** Cristina-Gabriela Grigoraș, Andrei-Ionuț Simion, Cătălin Drob

**Affiliations:** 1 Department of Food and Chemical Engineering, Faculty of Engineering, “Vasile Alecsandri” University of Bacău, Calea Mărășești 157, 600115 Bacău, Romania; 2 Department of Engineering and Management, Mechatronics, Faculty of Engineering, “Vasile Alecsandri” University of Bacău, Calea Mărășești 157, 600115 Bacău, Romania; catad@ub.ro

**Keywords:** chitosan, nanoparticle, hydrogel, dye, adsorption, isotherm models, kinetic models, thermodynamic studies, adsorbent recycling

## Abstract

Water is one of the fundamental resources for the existence of humans and the environment. Throughout time, due to urbanization, expanding population, increased agricultural production, and intense industrialization, significant pollution with persistent contaminants has been noted, placing the water quality in danger. As a consequence, different procedures and various technologies have been tested and used in order to ensure that water sources are safe for use. The adsorption process is often considered for wastewater treatment due to its straightforward design, low investment cost, availability, avoidance of additional chemicals, lack of undesirable byproducts, and demonstrated significant efficacious potential for treating and eliminating organic contaminants. To accomplish its application, the need to develop innovative materials has become an essential goal. In this context, an overview of recent advances in hydrogels based on chitosan and nanocomposites and their application for the depollution of wastewater contaminated with dyes is reported herein. The present review focuses on (i) the challenges raised by the synthesis process and characterization of the different hydrogels; (ii) the discussion of the impact of the main parameters affecting the adsorption process; (iii) the understanding of the adsorption isotherms, kinetics, and thermodynamic behavior; and (iv) the examination of the possibility of recycling and reusing the hydrogels.

## 1. Introduction

Water is one of the most important resources for both the environment and human survival. Nevertheless, due to the growing population, urban living, agricultural output, and intensive industrialization, the water quality is at risk. Over the years, severe contamination has been observed, and dyes are regularly among the reported persistent organic and mineral pollutants. Produced worldwide in impressive amounts and varieties [1], and classified depending on the materials sources (natural or synthetic), based on the chromophore groups’ (Table 1) or on auxochrome groups’ (Table 2) nature, dyes can easily end up in water where they can obstruct the light penetration, producing a major negative impact on the ecosystem due to a lower photosynthetic activity. Furthermore, dyes are extremely harmful and carcinogenic; their buildup in some aquatic species poses a significant environmental hazard as well as a risk to human health due to the possibility of skin irritation, allergic dermatitis, cancer, and mutations [2]. As a result, in order to guarantee that water supplies are not dangerous for use, many processes have been tried in various technologies applied for wastewater treatment. 

Of them, biological treatment techniques are based on the reduction of the wastewater’s biological oxygen demand concentration by bacterial cells feeding on organic molecules. The biological treatments function in aerobic or anaerobic conditions. In the aerobic treatment processes, because the implicated microorganisms use open air to integrate contaminants, the existence of oxygen is needed. In this situation, the noxious compounds are converted to carbon dioxide, water, and biomass. In opposition, the anaerobic treatment processes occur in the absence of oxygen because the used related microorganisms do not incorporate contaminants through free air. The contaminants are converted to carbon dioxide, methane, and biomass. Compared to the water entering the whole process, the resulting water is far cleaner [3]. Nonetheless, the material remaining after a biological treatment precipitates in settling tanks or is held as sludge on solid surfaces of plants, requiring supplementary steps for handling and storage.

Chemical techniques applied for wastewater treatment involve reactions through which contaminants are transformed into innocuous compounds. Advanced oxidation processes (AOPs) assisted by UV light, catalysts (usually metallic oxides), or oxidizing species (hydroxyl, sulfate, superoxide radicals etc.) are frequently used to transform the harmful pollutants into water and carbon dioxide [4]. AOPs consist of techniques such as electro-oxidation (direct or indirect), electro-coagulation, photocatalytic reactions, Fenton reactions, ozonation, etc. [5,6]. For instance, direct electro-oxidation involves the production of hydroxyl radicals, which can be absorbed chemically (creating various oxidation materials that aid in the breakdown of the pollutants) or physically (causing the complete decomposition of pollutant materials). In indirect electro-oxidation, hypochlorite produced by the anode and chlorine are combined [7]. This mixture aids in attacking the organic and inorganic constituents of the wastewater streams. In photocatalysis, which uses light and a semiconductor photocatalyst, in the initial step the target substrate is adsorbed on the semiconductor surface. Following the absorption of light with photon energy greater than the photocatalyst’s band-gap energy, photogenerated electron-hole pairs are created. A redox reaction and a recombination of certain photogenerated carriers from the surface and from the inside of the photocatalyst take place concomitantly as e^−^ and h^+^ move to the semiconductor surface. In a subsequent stage, water pollutants are broken down into small molecules while oxygen and water molecules are oxidized and reduced to hydroxyl and superoxide radicals. Ultimately, photoreaction persists as the broken-down molecules desorb from the interface to the main solution [8]. Fenton reactions are dependent on the electrochemical hydrogen peroxide produced and of its combination with the used catalyst in order to form hydroxyl radicals, which are responsible for the mineralization of the target molecules. Although the process has a limited working range, low conductivity, and low current density requirements, it is ecologically benign, economical, and requires basic equipment [9]. In ozonation processes, ozone may directly damage refractory compounds by electrophilic assault or indirectly through the attack of hydroxyl radicals produced during its breakdown. Ozonation reduces the amount of sludge that forms during wastewater treatment, but it also has the drawback of being difficult to adjust due to the ozone high instability and easy decomposition [10]. 

Physical treatments utilized for wastewater cleansing are realized through techniques such as membrane filtration (microfiltration, ultrafiltration, nanofiltration, reverse osmosis), ion exchange, coagulation/flocculation, flotation, adsorption, etc. Depending on the target pollutants, the types of membranes and the membrane cutoff size, the membrane filtration can be used at any point in the water treatment process. Through selective permeation, the membrane acts as a barrier to keep two phases apart. Electrical potential or a pressure difference may be the driving separation mechanism. The size of molecules and of membrane pores’ holes determine how the pollutants are separated [11,12]. Membranes are compact, simple and easy to operate and function without chemicals. They produce stable water and reduced amounts of sludge compared to other water treatment processes. At the same time, this type of technology implies elevated costs since it is energy-consuming and requires constant cleaning due to the high risk of fouling [13]. Coagulation/flocculation is particularly well-suited for wastewaters containing high levels of a broad range of different kinds of pollutants. Even though it is often thought to be a pre-treatment procedure, it is also feasible to lower the concentration of many pollutant species below the allowable limit. The process involves inorganic or organic coagulants. Usually sulfates or chlorides of aluminum and iron hydrolyze to neutralize the colloids that have a predominantly negative charge. Then, the impurities trapped in the metal hydroxide precipitates and sedimentation occur as a result [14]. Simple and cost-effective to function, the coagulation/flocculation needs considerable amounts of chemicals and leads to high quantities of sludge [15]. 

As seen above, the previously mentioned techniques (which can be equally applied for the treatment of wastewater containing dye residues) [16,17,18,19,20], aside from their benefits, also present major inconveniences, especially from the point of view of the implied high costs related to energy consumption and special operating conditions (the presence of specific chemicals, the assurance of a particular temperature, the existence or absence of oxygen, etc.).

Alternatively, an intensive focus is currently directed to the adsorption technique. Due to its simple design and low investment cost, because it is readily available, it avoids the additional chemicals, it does not lead to unwanted byproducts, and has been shown to have significant efficacious potential for treating and eliminating organic contaminants, this strategy is being strongly considered for wastewater treatment. Activated carbon is by far the most common material used in the processes [21,22], but many other adsorbents (derived from biomass [23,24], with magnetic properties [25], hybrid materials [26], composites [27], etc.) have been likewise prepared and researched to remove water pollutants. They are notable for the important surface area, consistent pore diameters, and for the functional groups capable of binding the emerging compounds. 

Many adsorption studies have been conducted also for the removal of dyes from aqueous matrices. Li et al. [28] synthesized a material based on chitosan-coated magnetic mesoporous silica nanoparticles and utilized it to efficiently eliminate Methylene blue from water. Jawad et al. [29] designed a new composite of chitosan grafted-benzaldehyde/ montmorillonite/algae and tested it against Brilliant green and Reactive blue 19 dyes. The recorded maximum adsorption capacities were 899.5 mg/g for Brilliant green and 213.6 mg/g for Reactive blue 19. The adsorption behavior of hydroxyapatite/graphene oxide/chitosan porous beads towards Methylene blue was the subject of the paper published by Hoa et al. [30]. They report an adsorption capacity of 99 mg/g. Akbarnejad et al. [31] prepared a magnetic chitosan/Al_2_O_3_/Fe_3_O_4_ nanocomposite and used it for the adsorption of Acid fuchsin dye. Promising results were obtained likewise by Zhang et al. [32], who generated an adsorbent of chitosan and phytic acid for the retention of Acid blue 2. Sayed et al. [33] casted thin films of chitosan and various proportion of erythritol, tested them as adsorbents for Methylene blue, and reported a maximum removal of 186.23 mg/g. Other conducted research [34,35,36,37] also reveals the possibility of using other different adsorbents for the favorable elimination of various dyes from aqueous solutions. Notwithstanding, finding new materials and technologies for wastewater management is still a challenging practice. It has become indispensable, and continuous efforts are being made in this area as concerns about water pollution intensify on a global scale.

Within this framework, the present paper is aimed to give an overview of the recent advances in employing hydrogels for water decontamination. The driving force for selecting this topic for assessment is given by the prevalence among the researchers as regards the development and the utilization of sustainable adsorption technology based on materials renowned for their ability to retain target chemicals. 

The attention of this review is directed to the hydrogels resulting from the combination of chitosan and nanoparticles and to their ability to adsorb pollutants from water. Firstly, the hydrogels’ synthesis and the methods used for a rigorous characterization will be addressed. Secondly, their application as adsorbents of dyes as model molecules will be discussed. The impact of different parameters influencing the adsorption process is analyzed. In addition, this review includes data about the adsorption process governing mechanisms and about the mathematical modeling in terms of kinetics, isotherms, and thermodynamics. It endeavors to also provide valuable information regarding the challenges of reusing hydrogels and their potential yet to come in applications in the field of wastewater treatment. 

## 2. Results and Discussion

### 2.1. Examination of the Encompassed Studies

The preferred reporting items for systematic reviews and meta-analyses (PRISMA) methodology was used for collecting the studies included in the present review. PRISMA offers a well-organized and transparent procedure for choosing pertinent research in addition to evaluating the results’ quality and summarizing them. Features such as certain research topics, publication dates, pertinent results, etc. that studies must have in order to be included in the review are outlined in the inclusion criteria. Conversely, exclusion criteria specify the grounds for rejecting investigations that do not fit the predetermined conditions [38]. 

**Figure 1:**
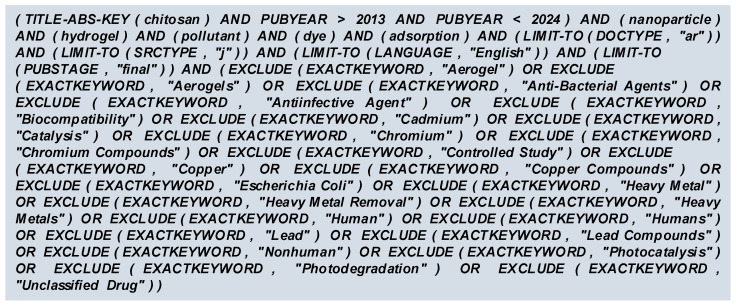
Search code used in Scopus database.

In the current review, several criteria both of inclusion and exclusion were used for conducting the process of paper screening in order to ensure that only the appropriate studies were taken into account. Therefore, a suitable search code has been written and supplied as an input in the Scopus database (Figure 1). 

With “chitosan” as the primary keyword, publications from the last full ten years (period comprising 2014 to 2023), declared as article papers and available in journals written in the English language in their final form were selected. Of a total of 109,987 records, only 52.28% met the above criteria. A refined search by adding the supplementary keywords “nanoparticle”, “hydrogel”, “pollutant”, “dye”, and “adsorption” revealed that only 1940 articles (1.76%) manuscripts seemed to be related to hydrogels based on chitosan and nanoparticles used for dyes adsorption from water.

The exploration of the keywords used in Scopus allowed the exclusion of 1066 records, which led to 874 potential documents. 

**Figure 2:**
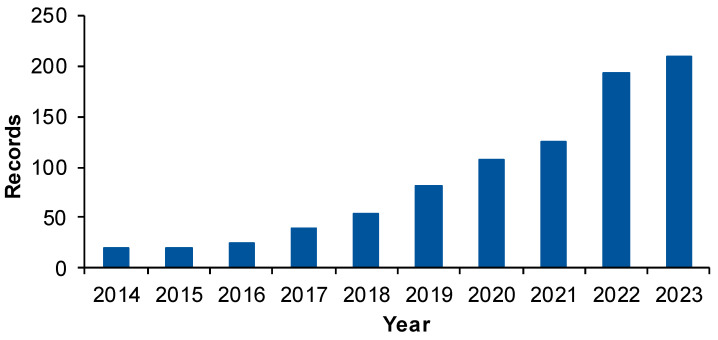
Scopus descriptive analysis of 874 records—records by year.

A graphical analysis of these records served to examine the publication behavior in the field. In terms of the number of records by year (Figure 2), it can be noted that in the first four years (2014–2018) of the considered period, only 105 papers were published and that an accelerated ascending trend can be observed between 2020–2023, with the number of records six times higher (634 documents). 

**Figure 3:**
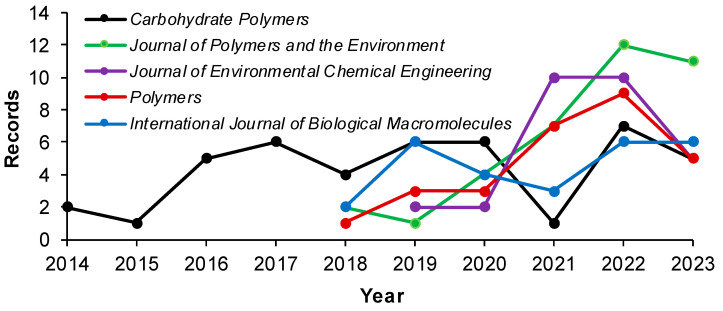
Scopus descriptive analysis of 874 records—records by year per source.

Figure 3 shows the first five journals including articles returned by Scopus by using the input search code. *Carbohydrate Polymers*, *Journal of Polymers and the Environment*, *Journal of Environmental Chemical Engineering*, *Polymers*, and *International Journal of Biological Macromolecules* published a varying numbers of documents. Only *Carbohydrate Polymers* reports records in each year from 2014 to 2023. The other four top journals began to publish in this field from 2018 to 2019. In the set period, a peak was reached in 2022 (44 records), while a slight decline can be seen in 2023 (32 records).

**Figure 4:**
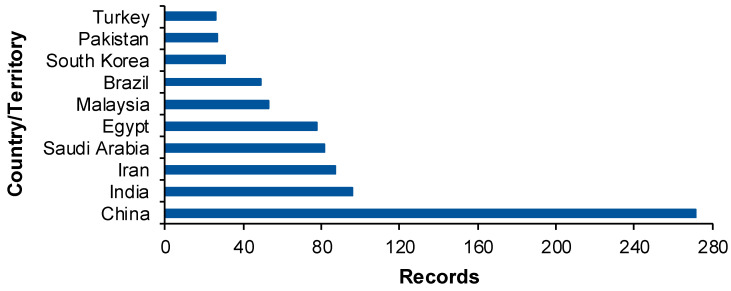
Scopus descriptive analysis of 874 records—records by country/territory.

The analysis of the records’ distribution by country/territory (Figure 4) shows that the top is China with 271 records. This could point to the existence of a correspondence between the population size and the conducted research. India, Iran, Saudi Arabia, Egypt, and Malaysia listed between 50 and 100 publications. Brazil, South Korea, Pakistan, and Turkey reported 25 to 49 articles in the period between 2014 and 2023. 

**Figure 5:**
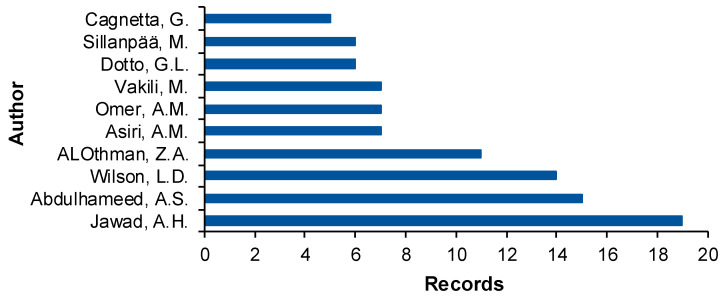
Scopus descriptive analysis of 874 records—records by author.

The top 10 authors who have written articles in the field in the last complete decade is given in Figure 5. Jawad, Abdulhameed, Wilson, and ALOthman published more than 10 papers each. Asiri, Omer, and Vakili published seven papers each. For Dotto and Sillanpaa six records each are listed. Finally, for Cagnetta five publications are reported. 

In the analysis of the 874 records returned by the Scopus database, an essential step was represented by the selection of the eligible articles for this review. A total of 503 publications (57.55%) were excluded after reading the titles, abstracts, and keywords. The full text of the remaining 371 articles was read and evaluated from the point of view of applicability, trustworthy results, valuable contribution, and relevance to practice. A total of 134 records (36.11%) were excluded due to the fact that the employed methodology was insufficiently described, the interpretation of the obtained results was unsatisfactory, or the contribution to the field was reduced or irrelevant. As a result, 203 documents were retained and carefully explored for this review. 

### 2.2. Synthesis and Charaterization of Hydrogels Based on Chitosan and Nanoparticles 

When looking for the best adsorbent, hydrogels have proven to be viable materials. Prominent qualities including the important surface area, high porosity, adsorption capacity stability, compatibility, and selectivity stand out, making them appropriate for removing water contaminants. Their capacity to absorb water insures a significant suppleness. The hydrophilic nature is responsible for their insolubility in solvents and for their ability to swell. The primary cause of this is the formed reticulated network in which the composing chains of macromolecules or their segments are linked either by permanent bonds or by more extensively arranged areas. 

Owing to characteristics such its biodegradability, biocompatibility, and lack of toxicity, chitosan has become a major component in the production of hydrogels. It results from chitin N-deacetylation, and it is made up of N-acetyl-p-glucosamine and β-(1→4)-p-glucosamine units that are dispersed randomly. Reactive hydroxyl and amino groups are present in chitosan’s structure, allowing for both chemical and physical changes. However, hydrogels containing only chitosan have several disadvantages, with poor mechanical strength, reduced thermal stability, and limited stability in acidic environments being among them. 

An approach capable of addressing these drawbacks consists of the integration of different nanoparticles into the hydrogels’ structure. 

Ren et al. [39] prepared chitosan microspheres loaded with silver nanoparticles and used them to adsorb Methylene blue from water. 

Abdelghaffar [40] explored the possibility of obtaining a nanocomposite derived from chitosan and silver nanoparticles. Firstly, the chitosan was dissolved in a glacial acetic acid solution. Then, a certain amount of a nanocomposite was added, and the mixture was sonicated. At the end, the suspension was washed and dried. When put in contact with a Reactive orange 5 dye solution, the material adsorbed 95% of the existing pollutant. 

Mohammad et al. [41] synthesized a cross-linked hydrogel by using a combination of chitosan glyoxal and titanium dioxide. The process was optimized by response surface methodology. The adsorbent retained 75.9% of the Methyl orange. 

In their study, Wei et al. [42] carried out an experimental program in which they mixed polyvinyl alcohol, multiwalled carbon nanotubes, chitosan, glacial acetic acid, glutaraldedyde, glycerol, and water to form a film hydrogel. The performance of the obtained material reached 101.07 mg/g when tested against Acid red 73 dye. 

Another research [43] present a hydrogel fabricated of chitosan dissolved in acetic acid with β-cyclodextin and cerium solution. Several hours of stirring was necessary and was followed by a cross-linking process with glutaraldehyde. The dried product was used for the adsorption of Reactive blue 4, Indigo carmine, and Acid blue 158, the adsorption capacities being 29.56 mg/g, 30.57 mg/g, and 30.57 mg/g, respectively. 

Solano et al. [44] reported that the beads made of chitosan, graphene oxide, and titanium dioxide nanoparticles removed 96.7% of Allura red AC dye tested as a target molecule. 

Mahmoud et al. [45] showed that the nanocomposite hydrogels synthesized by γ-radiation-induced copolymerization and cross-linking of chitosan, acrylic acid, and TiO_2_ nanoparticles successfully retained Methylene blue from the solution. 

Doondani et al. [46] applied a gelation method to obtain microspheres from magnetic chitosan/graphite/polyvinyl alcohol cross-linked with glutaraldehyde. Their experiments show that the adsorbent was able to remove 95% of the Reactive orange 16 dye. 

As seen from the studied literature, the preparation process of the hydrogels based on chitosan and nanoparticles is often conducted in several steps. Firstly, the biopolymer is dissolved in an acid solution (mainly glacial acetic acid) by stirring at room temperature to remove the air bubbles. In a second stage, the chosen nanoparticles (silver, titanium dioxide, iron oxide, cerium oxide, graphene oxide, silica, montmorillonite, activated carbon, hydroxyapatite, etc.) are added. After that, in some cases, a cross-linking step is applied, and glutaraldehyde is recurrently used to this purpose. The resulting mixture is frequently put in contact with a precipitation medium (represented by a salt solution). The formed hydrogels are used directly or after they have been dried in air or at different temperatures for retaining dyes molecules from aqueous solutions. 

In terms of hydrogel characterization, scanning electron microscopy (SEM), Fourier transform infrared spectrometry (FTIR), thermogravimetric analysis (TGA), X-ray diffraction analysis (XRD), X-ray photoelectron spectroscopy (XPS), transmission electron microscopy (TEM), zeta-potential measurements, determination of the point of zero charge (pH_PZC_), swelling behavior, ion exchange capacity, chemical stability, selectivity, etc. are cited as applied to this purpose. 

El-Kousi et al. [47] used chitosan and montmorillonite to prepare a hydrogel which was then modified by cross-linking with diethanol tartaramide and epichlorohydrin. For its characterization, they conducted an FTIR analysis, which confirmed the formation of the desired product. TGA analysis revealed the weight loss of the hydrogel depending on the temperature. XRD patterns served to indicate a disordered exfoliated structure. XPS evaluated the surface chemical changes and confirmed also the existence of montmorillonite nanoparticles in the chitosan matrix. SEM micrographs exposed the variations of the hydrogel surface before and after the adsorption of Methylene blue. 

In their study about the ability of the adsorbents based on chitosan and magnetic nanoparticles to remove Congo red dye from water, Klosert and her co-workers [48] used XRD analysis to investigate the presence of chitosan and of iron oxide nanoparticles in their product. The difference between the relative peaks’ intensities and the shifted positions, among others, sustained the idea that the chitosan was indeed coated with Fe_3_O_4_ nanoparticles. TGA and X-ray analyses along with the magnetic behavior of the adsorbent led to the same conclusion. 

For the characterization of the synthesized material from chitosan, sodium citrate, and activated charcoal, Nandanwar et al. [49] resorted also to FTIR spectra in a first step. The recorded vibrational peaks indicated the presence of charcoal and of the sodium citrate cross-linker in the obtained composite. X-ray diffractograms revealed supplementary peaks consistent with the existence of charcoal and of the cross-linker. TGA analysis contained a shift in exoterm to an elevated temperature, indicating the formation of the anticipated composite. Adsorption–desorption isotherms showed a porous nature attributed to the activated charcoal, while SEM images depicted an irregular and heterogeneous surface. 

Abdulhameed et al. [50] conducted a thorough analysis for the hydrogel prepared from chitosan loaded with different amounts of carbon-doped titanium dioxide. pH-potentiometric titration estimated the amino groups at 38.41%, suggesting that the obtained material can bind Methyl orange and Reactive orange 16 dyes. BET results indicated an important surface area, while TEM analysis accurately established the particles’ size. The functional groups were studied by FTIR before and after the adsorption of the chosen dye molecules and reiterated that the hydroxyl and amino groups participated in the process. The surface morphology was examined by SEM–EDX and revealed a granular surface with reduced pores. pH_PZC_ was equal to 7.0, signifying that the adsorbent is able to attract negative charges at pH values superior to pH_PZC_ and positive charges at pH values inferior to pH_PZC_. 

Kaur and Jindal [51] investigated the hydrogels resulting from a combination of chitosan, gelatin, and zirconium selenophosphate inorganic ion exchanger. The swelling studies showed a percentage of 508.061% at pH 7. The maximum ion exchange capacity was recorded for the organo-inorgano hybrid nanocomposite ion exchanger. The pH titration exhibited that the synthesized material was a solid cation exchanger. The contact with different chemical solutions indicated that the adsorbent possessed good stability in mineral acids at reduced concentration and that at high concentrations of acid, and in alkaline media, it dissociates. FTIR spectra, SEM, and TGA analyses confirmed the integration of chitosan and gelatin with the inorganic counterpart, while the conducted adsorption studies expressed very good retention of Methylene blue. 

Similar sets of analyses were carried out by other published research also [52,53,54,55,56,57,58,59]. This fact sustains the high significance of providing a careful, rigorous, and complete characterization of the new synthesized hydrogels, all the more so as it provides the necessary information to conduct the experiments regarding the adsorption of emergent pollutants (dyes included) from water in such a manner that in the adequate conditions, the decontamination process can be carried out efficiently and at low costs. 

### 2.3. Dyes Adsorption on Hydrogels Composed of Chitosan and Nanoparticles 

In order to scrutinize the ability of hydrogels prepared of chitosan and nanoparticles to eliminate pollutants from water, dyes were deliberately chosen as target compounds. The reason behind this choice stems from the fact that, even at low concentrations, these persistent organic pollutants represent a serious hazard and were identified as being present in aqueous media. Exposure to these types of complex compounds may have unpleasant consequences for humans, flora, fauna, and aquatic organisms. Negative impacts include carcinogenic, mutagenic, neurotoxic, and teratogenic impacts, with major health threats comprising intellectual impairment in youngsters and sleeplessness, and different organ malfunctions have been reported [60,61]. 

Table 3 summarizes some of the records retained for the present review regarding the prepared adsorbent materials, the dyes on which they were tested, and the experimental conditions along with the isotherm and kinetic models describing the process. 

One can note that the authors selected one dye model molecule (regularly) or a combination of two or more such molecules (less frequently) and that the working parameters were represented by the initial concentrations of the adsorbate and of the adsorbent, the pH, the temperature, the contact time, and the stirring speed. Most of the articles studied the influence of each factor separately, but there are also papers in which optimization processes were carried out by response surface methodology [36,62,63] or with the help of artificial neural network [64]. This sub-section includes appreciation of the influence of pH, of the employed adsorbent quantity, and of the stirring speed. The consequences of the other parameters (dye initial concentration, time, temperature) will be debated in the following dedicated sub-sections. 

pH is among the first set parameters. For example, Sadiq et al. [65], who prepared a hydrogel of chitosan, magnetic nanoparticles, and deep eutectic solvents and tested it on the adsorption of Malachite green, appraised first of all the impact of pH on the process. Their results show that the optimum pH is 4. Below that value, a repulsion of the amino groups existing on the adsorbent surface led to reduced adsorption. The retention is also diminished at a pH higher than the pH_PZC_, a fact explained by the abundance of hydroxyl ions in the alkaline media, which concur with dye ions to the adsorbent active sites. The effect of the adsorbent amount was favorable, and a removal percentage of 96.60% was reported. However, an excessive mass of adsorbent is detrimental and explained by the high availability of unsaturated sites for dye adsorption. 

Simonescu et al. [66] performed a comparative study to eliminate Congo red and Methyl orange from single and combined solutions by adsorption on a fixed amount of cobalt ferrite—chitosan hydrogel. For Congo red, the recommended pH was 10.8, while for Methyl orange, the value reached only 2.22. From the data reported for binary solutions, a decrease in the adsorption capacity was observed, suggesting that dyes may have an inhibitory effect one on the other. 

Santillan et al. [62] synthesized magnetic chitosan beads and introduced them into aqueous solutions of Synozol red and Synozol yellow. They included extreme acid and basic pH and noticed that efficient dyes removal takes place at pH 2 due to the electrostatic interactions between the amino groups of the adsorbent and the sulfonic groups of the target molecules. When the pH increases, fewer amino groups are available, and the adsorption declines. The concentration of the adsorbent was between 1 g/L and 8 g/L. The appropriate value was set at 2 g/L for both dyes. The stirring speed was varied from 50 rpm to 200 rpm, and the results showed that it enhances the adsorption. 

Abdulhameed et al. [63] prepared an adsorbent of chitosan and nanoparticles of titanium dioxide and used it for removing Reactive red 4 dye from an aqueous solution. They optimized the adsorption process by developing a Box–Behnken design with pH (4–10), adsorbent dose (0.5–1.5 g/L), and contact time (30–90 min) as input parameters. The highest value for the output function (removal rate) was obtained after 60 min, at pH 4, with 1.5 g/L adsorbent. 

Muangrak et al. [67] formulated an adsorbent by using montmorillonite and chitosan and tried its performance with respect to eliminating Reactive red 120. pH modification from 3 to 10 changed the material adsorption capacity. The hydrophobic interaction and electrostatic attraction between dye and the adsorbent occasioned the complete dye removal both from acidic and from basic media but with different adjustments of contact time. The influence of the adsorbent amount was also evaluated. The Reactive red 120 was totally removed by 1.2 g of material after a period of 90 min. 

Batch adsorption studies were carried out also by Patel et al. [68] with chitosan-(acrylic acid-co-(3-acrylamidopropyl) trimethylammonium chloride-co-(1,1-diallyl-4-carboxypiperidin-1-ium bromide))/Fe_3_O_4_ as adsorbent and Indigo carmine and Congo red dyes as emergent organic water pollutants. The pH varied from 1 to 12 and the adsorbent from 0.005 g to 0.025 g. The fact that the hydrogel contained ionizable carboxyl and amino groups led to an increase in the adsorption capacity. 

In their research conducted for 14 h, under shaking (100 rpm) with chitosan–quartzite adsorbent having a concentration of 2.5 g/L and Reactive black 5 dye with a concentration of 70 mg/L, Coura et al. [69] varied the pH between 4 and 10. The optimum value was set at 5 in these conditions, with the removal reaching 69.66%. 

Da Silva et al. [70] synthesized a hydrogel of polyacrylamide grafted on chitosan, cross-linked with N,N-methylenebisacrylamide and used it to adsorb Acid blue 113 dye. The adsorbent concentration and the stirring speed were kept constant at 0.6 g/L and 100 rpm, respectively. They monitored the effect of pH (2–10) and remarked that the adsorbent preparation procedure had a crucial impact on the results. When the material was obtained by the classical method, the recommended pH was found to be 6, while when the microwave-assisted synthesis was employed, the suggested pH was 2. They explained that in the first case, at low pH, the sulfonate groups of the tested molecule are protonated causing a diminishment of the attraction between the chitosan surface positively charged and the dye. At alkaline pH, when both the dye and the chitosan have negative charges, a repulsion effect and a reduced removal efficiency can be noted. In the second case, they concluded that the main interaction of the hydrogel with the dye is not an electrostatic but rather an ionic one. They did not exclude the sorption on surface groups, the physical sorption, and dipole–dipole hydrogen bonding. 

Another study [71] with a constant adsorbent dose and without stirring was conducted with a chitosan hydrogel cross-linked with trimellitic anhydride isothiocyanate and filled with single-walled carbon nanotubes and with Basic red 12 dye showed that the maximum adsorption capacity was registered at pH 12, and the lowest one was obtained at pH 4, which was consistent with the predominant charge of the adsorbent. 

Vakili et al. [72] prepared a hexadecylamine-impregnated chitosan-activated carbon adsorbent. In their report, Reactive black 5 was the target aqueous compound. The effect of the adsorbent dose (10–50 g/L) was examined. Up to a concentration of 30 g/L, the increased surface area accompanying the increased adsorbent amount led to an augmentation of the removal efficiency. Above this concentration, due to a possible agglomeration of the adsorbent particles, a decrease in dye retention was seen. As in other studies, the adsorption was also dependent on the dye solution initial pH maximum uptake being recorded at pH 4.

**Table 3:** Summary of some records included in the review regarding the synthesized hydrogels and their ability to adsorb dye molecules.

Adsorbent	Dye	Working Conditions *	Removal Efficiency	Adsorption Capacity	Isotherms Models	Kinetics Models	Reference
Chitosan/2-mercaptobenzimidazole	Methylene blue	1. 100 mg/L 2. 0.5–5 g/L 3. 1–11 4. 25–55 °C 5. 0–90 min 6. 50–300 rpm	92%	1.28 mmol/g	Langmuir Freundlich Sips	Pseudo-first-order Pseudo-second-order Intraparticle diffusion	[73]
Chitosan/clinoptilolite	Methyl orange	1. 35.34–282.35 mg/L 2. 1–12 g/L 3. 2.2–10 4. 30 °C 5. 0–40 min 6. No stirring	77.23%	16.88 mg/g	Langmuir Freundlich Redlich–Peterson Toth Sips	Not available	[74]
Chitosan/N-(3-dimethylaminopropyl)-N′-ethylcarbodiimide hydrochloride/cysteine	Methylene blue (MB) Methyl orange (MO)	1. 0–100 mg/L 2. 2600 mg/L 3. 7 4. 23 °C 5. 0–1440 min 6. No stirring	MB 30% MO 91%	MB 115 mg/g MO 305 mg/g	Langmuir Freundlich	Pseudo-second-order Pseudo-first-order	[75]
Chitosan/cellulose	Malachite green	1. 50–200 mg/L 2. 6.66 g/L 3. Natural 4. Ambient 5. 0–45 min 6. No stirring	98.65%	115.1 mg/g	Langmuir Freundlich	Pseudo-second-order Pseudo-first-order Intraparticle diffusion	[76]
Chitosan-salicylaldehyde Schiff base/algae/montmorillonite	Remazol brilliant blue R (RBR) Brilliant green (BG)	1. 20–300 mg/L RBR, 20–200 mg/L BG 2. 0.2–0.8 g/L 3. 4–9 4. Ambient 5. 5–55 min 6. Under stirring	RBR 54.3% BG 79.4%	RBR 148.1 mg/g BG 440.3 mg/g	Freundlich Langmuir	Pseudo-second-order	[77]
Iron (III) hydroxide/chitosan	Alizarin red S	1. 50 mg/L 2. 0.01–0.9 g/L 3. 5 4. 30–80 °C 5. 0–360 min 6. 200 rpm	Not available	294 mg/g	Langmuir	Pseudo-second-order Pseudo-first-order	[78]
EDTA/chitosan/magnetic graphene oxide nano-sheets	Rhodamine B	1. 50–250 mg/L 2. 0.07–0.18 mg/L 3. 4–9 4. 20–50 °C 5. 5 min 6. Under shaking	92%	1085.3 mg/g	Langmuir Freundlich Temkin	Pseudo-second-order Elovich Intraparticle diffusion Pseudo-first-order	[79]
Carboxymethyl β-cyclodextrin/nanochitosan/glutaraldehyde	Acid red 37	1. 80–214 mg/L 2. 0.16 –1 g/L 3. 2–9.3 4. 20–40 °C 5. 0.33–10 min 6. 250 rpm	99.6%	332.60 mg/g	Langmuir Freundlich Temkin Flory–Huggins Dubinin–Radushkevich	Pseudo-second-order Pseudo-first-order Elovich Intraparticle diffusion	[80]
Magnetic polyethyleneimine nanoparticles/sulfonated chitosan/glutaraldehyde.	Methylene blue	1. 100 μmol/L 2. 2.5 g/L 3. Natural 4. Ambient 5. 0–360 min 6. Under stirring	≈50%	Not available	Not available	Not available	[81]
Gelatin/chitosan/β-cyclodextrin/sodium humate	Methylene blue (MB) Acid fuchsin (AF)	1. 100–3000 mg/L 2. 1 g/L 3. 2–10 4. 15–50 °C 5. 0–300 min 6. 200 rpm	Not available	MB 1666.7 mg/g AC 714.3 mg/g	Langmuir Freundlich Temkin	Pseudo-second-order Pseudo-first-order Intraparticle diffusion	[82]
Graphene oxide/chitosan/magnetic nanoparticles	Sudan I, II, III, IV (SI, SII, SIII, SIV)	1. 100–400 mg/L 2. 0.1–1 g/L 3. 2–10 4. 15–45 °C 5. 30–120 min 6. 150 rpm	>90%	SI 360.6 mg/g SII 353.7 mg/g SIII 351.0 mg/g SIV 347.6 mg/g	Langmuir Hill Freundlich Temkin Redlich–Peterson	Pseudo-second-order Elovich Pseudo-first-order Intraparticle diffusion	[83]
Magnetite nanoparticles/amino-silane/graphene oxide/chitosan/diethylenetriaminepentaacetic acid	Methyl violet	1. 8–30 mg/L 2. 0.05–3.7 g/L 3. 2–12 4. 13.1–71.9 °C 5. 30–120 min 6. 150 rpm	94.87%	243.8 mg/g	Sips Modified Langmuir–Freundlich Langmuir Extended Langmuir Redlich–Peterson Temkin Toth	Pseudo-first-order Pseudo-second-order Elovich Mixed 1, 2-order Pseudo n-order Fractal-like pseudo-first-order Fractal-like pseudo-second-order	[84]
Montmorillonite/ chitosan	Calmagite (C) Methylene blue (MB)	1. 50 mg/L 2. 0.3 g/L 3. 4–10 4. Ambient 5. 0–1800 min 6. No stirring	C ≈ 80% MB 75%	Not available	Not available	Pseudo-second-order	[85]
Amphoteric chitosan/gelatin	Acid red 337	1. 50–500 mg/L 2. 0.5 g/L 3. 1–10 4. 20–50 °C 5. 1–700 min 6. 100 rpm	95.6%	≈750 mg/g	Langmuir Dubinin–Radushkevich Freundlich	Pseudo-second-order Intraparticle diffusion Pseudo-first-order	[86]
Gelatin/chitosan/β-cyclodextrin	Malachite green (MG) Crystal violet (CV) Congo red (CR) Methylene blue (MB) Acid fuchsin (AF) Methyl orange (MO)	1. 100–2800 mg/L 2. 0.5 g/L 3. 2–10 4. 15–50 °C 5. 0–240 min 6. 200 rpm	MG 80% CV 83.3% CR 88.9% MB 85.1% AF 93.3% MO 87.5%	MG not available CV not available CR not available MB 667 mg/g AF 1111 mg/g MO not available	Langmuir Freundlich Temkin	Pseudo-second-order Pseudo-first-order Intraparticle diffusion Film diffusion	[87]
Chitosan/montmorillonite	Methyl orange	1. 20–320 mg/L 2. 0.32–1.92 mg/cm^2^ 3. 4–11 4. 30–60 °C 5. 1–60 min 6. No stirring	96.2%	154.4 mg/g	Langmuir Freundlich Temkin	Pseudo-second-order Pseudo-first-order	[88]
Graphene oxide/chitosan	Congo red	1. 300–600 mg/L 2. 0.25 g/L 3. 3–12 4. 27–60 °C 5. 3–60 min 6. 200 rpm	Not available	1666 mg/g	Langmuir Freundlich Temkin	Pseudo-second-order Pseudo-first-order Intraparticle diffusion	[89]
CaNiFe_2_O_4_/ chitosan	Methylene blue	1. 100–500 mg/L 2. 1–10 g/L 3. 2–12 4. 25–65 °C 5. 5–300 min 6. 400 rpm	Not available	700 mg/g	Langmuir Freundlich Dubinin–Radushkevich	Pseudo-second-order Pseudo-first-order Intraparticle diffusion	[90]
Chitosan/β-Cyclodextrin	Indigo carmine	1. 50–200 mg/L 2. 0.1–1 g/L 3. 3–6 4. 15–50 °C 5. 5–300 min 6. No stirring	≈100%	1000 mg/g	Langmuir Freundlich	Pseudo-second-order Pseudo-first-order Intraparticle diffusion	[91]
Tamarind seed-activated carbon/chitosan	Methylene blue (MB) Methyl orange (MO)	1. 5–50 mg/L 2. 1–3 g/L 3. 2–12 4. Ambient 5. 0–120 min 6. No stirring	Not available	MB 140.62 mg/g MO 94.45 mg/g	Langmuir Freundlich Temkin Dubinin–Radushkevich	Pseudo-second-order Pseudo-first-order Intraparticle diffusion Elovich	[92]
Chitosan/montmorillonite	Reactive red 136	1. 50–400 mg/L 2. 0.3–0.6 g/L 3. 3–6 4. 20–50 °C 5. 54–180 min 6. 150 rpm	74.7%	445.38 mg/g	Toth Sips Langmuir–Freundlich Langmuir Radke–Prausnitz Dubinin–Radushkevich	Fractal-like mixed 1,2 order Mixed 1,2 order Pseudo-second-order Fractal-like pseudo-2nd-order Fractal-like pseudo-1st-order Pseudo-first-order Elovich	[93]
Nano chitosan/activated carbon	Rose Bengal	1. 1–7 mg/L 2. 1 g/L 3. 6.5–9.5 4. 22–50 °C 5. 0–120 min 6. 150 rpm	94.7%	Not available	Langmuir Freundlich	Pseudo-second-order Pseudo-first-order Liquid film diffusion Intraparticle diffusion	[94]
Rice bran/chitosan/aniline (RBCA) Rice bran/chitosan/pyrrole (RBCP)	Malachite green	1. 5–200 mg/L 2. 0.05–0.3 g/L 3. 2–9 4. 30–60 °C 5. 5–120 min 6. 120 rpm	Not available	RBCA 145.03 mg/g RBCP 55 mg/g	Freundlich Langmuir Temkin	Pseudo-second-order Pseudo-first-order Intraparticle diffusion	[95]
Chitosan/Fe_3_O_4_ (MCMs); chitosan/Fe_3_O_4/_ [poly(2-(dimethylamino)ethyl methacrylate)] (GMCMs)	Acid green 25 (AG25) Reactive blue 19 (RB19)	1. 1600 mg/L AG25; 1400 mg/L RB19 2. 1 g/L 3. 4–7 4. 30 °C 5. 0–420 min 6. No stirring	Not available	MCMs AG25 411.9 mg/g RB19 137.8 mg/g GMCMs AG25 961.5 mg/g RB19 691.3 mg/g	Langmuir Freundlich	Pseudo-second-order Pseudo-first-order	[96]
Chitosan/tripolyphosphate	Basic blue 7	1. 50–600 mg/L 2. 0.3–1.2 g/L 3. 2–8 4. 25–55 °C 5. 0.5–390 min 6. No stirring	100%	1174 mg/g	Langmuir Fowler–Guggenheim Freundlich Temkin	Pseudo-second-order Pseudo-first-order	[97]
Chitosan/Fe_3_O_4/_graphene oxide	Eriochrome black T (EBT) Methylene blue (MB)	1. 250–400 mg/L 2. 1 g/L 3. 2–8 4. 25–45 °C 5. 20–180 min 6. 150 rpm	EBT 86.67% MB 73.33%	EBT 289.85 mg/g MB 261.78 mg/g	Langmuir Freundlich	Pseudo-second-order Pseudo-first-order	[98]
Chitosan/clinoptilolite	Methyl violet	1. 25–125 mg/L 2. 0.5–10 g/L 3. 2–9 4. 25–45 °C 5. 5–120 min 6. 250 rpm	Not available	111.11 mg/g	Langmuir Freundlich	Pseudo-second-order Pseudo-first-order	[64]
Chitosan/benzil	Reactive orange 16	1. 20–200 mg/L 2. 0.02–0.08 g 3. 4–10 4. 25–45 °C 5. 5–25 min 6. 250 rpm	98.2%	291.8 mg/g	Freundlich Langmuir	Pseudo-second-order Pseudo-first-order	[99]
Chitosan/sodium alginate/graphene oxide Chitosan/sodium alginate/graphene oxide/β-cyclodextrin	Rose Bengal	1. 2–12 mg/L 2. 0.05–0.25 g/L 3. 2–13 4. 30–70 °C 5. 0–660 min 6. Under agitation	>90%	Not available	Langmuir Elovich Freundlich Dubinin–Radushkevich Temkin	Pseudo-second-order Elovich Pseudo-first-order Intraparticle diffusion	[100]
Zeolitic imidazole framework/chitosan	Congo red (CR) Malachite green (MG)	1. 1–50 mg/L 2. 0.02 g/L 3. 4–11 4. 25–45 °C 5. 0–180 min 6. No stirring	Not available	MG 384.6 mg/g CR 500 mg/g	Langmuir Redlich–Peterson Freundlich Temkin	Pseudo-first-order Pseudo-second-order Elovich Intraparticle diffusion	[101]
Graphene oxide/chitosan	Reactive black 5	1. 100–600 mg/L 2. 0.033–1.66 g/L 3. 2–11 4. 30–70 °C 5. 1–90 min 6. 50–250 rpm	98.66%	638.93 mg/g	Langmuir Temkin Freundlich	Pseudo-second-order Pseudo-first-order Elovich Intraparticle diffusion	[102]
Rice bran/chitosan	Reactive blue 4	1. 200 mg/L 2. 0.5–3.0 g/L 3. 2–10 4. 30–60 °C 5. 0–600 min 6. No stirring	60%	57 mg/g	Langmuir Freundlich	Intraparticle diffusion Pseudo-first-order Pseudo-second-order	[103]
Chitosan/graphene oxide	Reactive blue 19	1. 20–60 mg/L 2. 0.1–1.5 g/L 3. 4–9 4. 20–50 °C 5. 10–120 min 6. No stirring	99%	Not available	Freundlich Langmuir	Pseudo-second-order Pseudo-first-order	[104]
Fe_3_O_4/_chitosan	Acid blue	1. 50–1000 mg/L 2. 1 g/L 3. 3–11 4. 30 °C 5. 0–720 min 6. 175 rpm	80%	142 mg/g	Langmuir	Pseudo-second-order Pseudo-first-order	[105]
Polyethylene glycol-/graphene oxide/chitosan	Methyl orange	1. 200–25000 mg/L 2. 1 g/L 3. 2–10 4. Ambient 5. 5–90 min 6. 200 rpm	Not available	150 mg/g	Langmuir Freundlich	Pseudo-second-order Pseudo-first-order	[106]
Chitosan/cetyltrimethylammonium bromide-aliquat-366	Tartrazine	1. 160–3000 mg/L 2. 2 g/L 3. 4–11 4. 25–45 °C 5. 0–45 min 6. No stirring	90.36%	45.95 mg/g	Langmuir Freundlich	Pseudo-second-order Pseudo-first-order	[107]
Chitosan/graphene oxide/copper ferrite	Safranin O (SO) Indigo carmine (IC)	1. 1 × 10^−5^–1 × 10^−4^ M 2. 0.1–0.4 g/L 3. 2–10 4. 20–35 °C 5. 10–100 min 6. 400 rpm	SO 96% IC 95.91%	SO 66.15 mg/g IC 112.6 mg/g	Langmuir Freundlich Temkin Sips Redlich–Peterson	Pseudo-second-order Pseudo-first-order Intraparticle diffusion Elovich	[108]
Chitosan/Schiff base pyrano [3,2-c]quinoline-3-carboxaldehyde	Remazol red	1. 10–100 mg/L 2. 1 g/L 3. 3–11 4. 20–40 °C 5. 0–60 min 6. No stirring	100%	344.8 mg/g	Langmuir Temkin Dubinin–Radushkevich	Pseudo-second-order Pseudo-first-order Intraparticle diffusion Elovich	[109]
Magnetic calcium/chitosan	Orange II (OII) Methylene blue (MB)	1. 30–400 mg/L 2. 0.2 g/L 3. 2–11 4. 4–40 °C 5. 0–1440 min 6. No stirring	90%	OII 492 mg/g MB 350 mg/g	Langmuir Temkin Freundlich	Pseudo-second-order Pseudo-first-order Intraparticle diffusion	[110]
N-Guanidinium/chitosan/silica	Methyl orange	1. 25–2500 mg/L 2. 0.05 g/L 3. 2–9 4. 25 °C 5. 10–160 min 6. No stirring	95%	917 mg/g	Langmuir Freundlich	Pseudo-second-order Pseudo-first-order	[111]
Chitosan/fly ash/Fe_3_O_4_	Reactive orange 16	1. 20–150 mg/L 2. 0.04–0.12 g/L 3. 4–10 4. 30–50 °C 5. 0–660 min 6. No stirring	73.1%	66.9 mg/g	Freundlich Langmuir Temkin	Pseudo-second-order Pseudo-first-order	[112]
Graphene oxide/chitosan/polyvinyl alcohol	Congo red	1. 10–25 mg/L 2. 1–6 g/L 3. 2–8 4. 25–45 °C 5. 10–150 min 6. 140 rpm	88.17%	2.4 mg/g	Langmuir Freundlich	Pseudo-second-order Pseudo-first-order	[113]
Chitosan/magnetic metal-organic framework	Congo red	1. 50–600 mg/L 2. 5–30 mg 3. 6–12 4. 25–45 °C 5. 0–250 min 6. 250 rpm	99.8%	310.4 mg/g	Liu Langmuir Freundlich	Pseudo-second-order Pseudo-first-order	[114]
Chitosan/polyvinyl alcohol/MgO/Fe_3_O_4_	Remazol brilliant blue R	1. 20–240 mg/L 2. 0.2–1 g/L 3. 4–10 4. 30–60 °C 5. 0–180 min 6. No stirring	63.5%	163.7 mg/g	Freundlich Langmuir Temkin	Pseudo-second-order Pseudo-first-order	[115]
Chitosan/hydroxyapatite	Crystal violet	1. 10–400 mg/L 2. 0.4–4 g/L 3. 3–12 4. 25–65 °C 5. 5–180 min 6. 100–700 rpm	93.21%	Not available	Redlich–Peterson Langmuir Freundlich Temkin	Pseudo-second-order Pseudo-first-order Intraparticle diffusion Elovich	[116]
Polypyrrole/chitosan/graphene oxide	Ponceau 4R	1. 2.4–4.32 mg/L 2. 0.16–0.66 g/L 3. 2–12 4. 30–45 °C 5. 2–210 min 6. 200 rpm	92.16%	6.277 mg/g	Langmuir Freundlich Temkin Dubinin–Radushkevich	Pseudo-second-order Pseudo-first-order Intraparticle diffusion	[117]
Magnetic halloysite/chitosan	Methylene blue	1. 20–200 mg/L 2. 1 g/L 3. 5–11 4. 25 °C 5. 0–1440 min 6. 20 rpm	83.9%	50.37 mg/g	Langmuir Freundlich Redlich–Peterson	Pseudo-second-order Pseudo-first-order	[118]
Chitosan Schiff base (chloroethanoic acid, isopropyl alcohol)	Bismarck brown R (BBR) Eosin Y (EY)	1. 50–100 mg/L 2. 1–3 g/L 3. 2–10 4. 30–40 °C 5. 10–120 min 6. No stirring	BBR 94.5% EY 99%	BBR 327 mg/g EY 386 mg/g	Langmuir Freundlich Temkin Dubinin–Radushkevich	Pseudo-second-order Pseudo-first-order	[119]
Graphene oxide/chitosan/MnO_2_	Amido black 10B (AB10) Methylene blue (MB)	1. 10–200 mg/L 2. 0.6 g/L 3. 2–12 4. 25 °C 5. 0–800 min 6. No stirring	AB10 97% MB 80%	AB10 120 mg/g MB 320 mg/g	Langmuir Freundlich	Pseudo-second-order Pseudo-first-order Intraparticle diffusion	[120]
Iron oxide/polyvinyl alcohol/chitosan combined/activated carbon	Methylene blue	1. 0.015–0.025 mg/L 2. 0.6 g/L 3. 7 4. Ambient 5. 0–310 h 6. No stirring	Not available	22.4 mg/g	Langmuir Freundlich	Pseudo-second-order Pseudo-first-order Elovich Intraparticle diffusion	[121]
Iron oxide/polyvinyl alcohol/chitosan combined/activated graphite	Methylene blue	1. 15–25 mg/L 2. 0.6 mg/L 3. 7 4. Ambient 5. 13 days 6. No stirring	53.962%	36.385 mg/g	Not available	Pseudo-second-order Pseudo-first-order Elovich	[122]
Zinc oxide/chitosan	Eriochrome black T	1. 20–100 mg/L 2. 0.1–1 g/L 3. 2–12 4. Ambient 5. 0–120 min 6. No stirring	92%	40.9 mg/g	Langmuir Freundlich Temkin	Pseudo-first-order Intraparticle diffusion	[123]
Kaolin/Chitosan/ TiO_2_	Crystal violet	1. 20–60 mg/L 2. 0.5–2 g/L 3. Natural 4. 25–45 °C 5. 0–180 min 6. 250 rpm	93.30%	Not available	Freundlich Temkin Langmuir	Pseudo-second-order Pseudo-first-order Elovich Power function	[124]
Chitosan/sodium alginate/graphene oxide (CH-ALG-GO); Chitosan/sodium alginate/bentonite (CH-ALG-BN)	Xylenol orange (XO) Methylene blue (MB)	1. 2–10 mg/L 2. 25 g/L 3. 2–13 4. 30–70 °C 5. 0–540 min 6. No stirring	CH-ALG-GO XO 85% MB 91% CH-ALG-BN XO 93% MB 96%	CH-ALG-GO Not available CH-ALG-BN XO 0.195 mg/g MB 0.731 mg/g	Langmuir Freundlich Elovich Temkin	Pseudo-second-order Elovich Intraparticle diffusion Pseudo-first-order	[125]
Chitosan/talc/cloisite 30B clay	Crystal violet (CV) Reactive yellow 145 (RY)	1. 10–40 mg/L CV; 30–60 mg/L RY 2. 0.1–1.25 g/L 3. 4–10 4. Ambient 5. 0–240 min 6. No stirring	Not available	CV 37.03 mg/g RY 76.9 mg/g	Langmuir Freundlich Dubinin–Radushkevich Temkin	Pseudo-second-order Pseudo-first-order Intraparticle diffusion Elovich	[126]
Chitosan/hydroxyapatite	Titan yellow (TY) Reactive blue 4 (RB4)	1. 50–2000 mg/L TY; 50–2500 mg/L RB4 2. 3 g/L 3. 4–10 4. 25–55 °C 5. 0–160 min 6. 300 rpm	Not available	TY 170.7 mg/g RB4 118.4 mg/g	Langmuir Freundlich Sips Extended Langmuir Extended Freundlich	Pseudo-second-order Pseudo-first-order Intraparticle diffusion	[127]
Chitosan/poly-(itaconic acid-co-diallyl dimethyl ammonium chloride)/Fe_3_O_4_	Congo red (CR) Methylene blue (MB)	1. 100–500 mg/L 2. 0.005–0.025 g/L 3. 2–12 4. 25 °C 5. 0–100 min 6. No stirring	Not available	CR 862.06 mg/g MB 1111.11 mg/g	Freundlich Langmuir	Pseudo-second-order Pseudo-first-order	[128]
Chitosan/silica	Food green 3	1. 30–400 mg/L 2. 1–5 g/L 3. 3–9 4. Ambient 5. 5–60 min 6. 600 rpm	99.31%	476.19 mg/g	Langmuir Freundlich	Pseudo-second-order	[129]
α-ketoglutaric acid Schiff base/chitosan	Congo red	1. 50–300 mg/L 2. 250–1250 mg/L 3. 3–11 4. 25–55 °C 5. 0–60 min 6. No stirring	94.87%	434.78 mg/g	Dubinin–Radushkevich Langmuir Freundlich	Pseudo-second-order Pseudo-first-order Elovich	[130]
Kaolin/chitosan	Congo red	1. 25–200 mg/L 2. 2–12 g/L 3. 4–10 4. 25–55 °C 5. 1–180 min 6. 50–250 rpm	97%	104 mg/g	Freundlich Langmuir Dubinin–Radushkevich	Pseudo-first-order Pseudo-second-order Intraparticle diffusion	[131]
Chitosan/magnesium oxide	Methyl orange	1. 10–30 mg/L 2. 0.1–0.5 g/L 3. 6–10 4. 10–50 °C 5. 0–30 min 6. Under agitation	96.42%	237.5 mg/g	Langmuir Redlich–Peterson Temkin Freundlich	Pseudo-second-order Pseudo-first-order Elovich Intraparticle diffusion	[132]
Chitosan/zero-valent iron	Direct red 81	1. 10–50 mg/L 2. 0.05–2 g/L 3. 3–9 4. 15–55 °C 5. 2–12 min 6. No stirring	97%	Not available	Freundlich Langmuir	Pseudo-first-order Pseudo-second-order	[133]
Polyacrylamide-g/chitosan/γ-Fe_2_O_3_	Malachite green	1. 15–75 g/L 2. 0.5–1.5 g 3. 3–7 4. 25–45 °C 5. 80–210 min 6. No stirring	73%	86.28 mg/g	Langmuir Freundlich Temkin Dubinin–Radushkevich	Pseudo-first-order Pseudo-second-order Elovich Intraparticle diffusion	[134]
Chitosan/choline chloride/urea/FeO	Acid blue 80	1. 25–250 mg/L 2. 1–7 g/L 3. 3–10 4. 25–45 °C 5. 15–480 min 6. 350 rpm	99.30%	61.64 mg/g	Langmuir Freundlich Temkin Dubinin–Radushkevich Elovich	Pseudo-second-order Pseudo-first-order Intraparticle diffusion Elovich	[135]
Chitosan/polypropenoic acid/ethylenediamine/magnetite	Astrazon blue (AB) Lerui acid brilliant blue (LA)	1. 100–400 mg/L 2. 4 g/L 3. 3–11 4. 25–45 °C 5. 30–1440 min 6. 100 rpm	AB 80% LA 40%	AB 193.21 mg/g LA 51.90 mg/g	Langmuir Freundlich Elovich Temkin Dubinin–Radushkevich Harkin–Jura	Pseudo-first-order Pseudo-second-order Intraparticle diffusion	[136]
Diethylenetriamine/chitosan/Fe_3_O_4/_Cu	Methyl orange	1. 10–100 mg/L 2. 0.5 g/L 3. Natural 4. 25 °C 5. 0–40 min 6. No stirring	96.40%	144.60 mg/g	Langmuir Freundlich	Pseudo-second-order Pseudo-first-order	[137]
Chitosan/MgO/Fe_3_O_4_	Reactive blue 19	1. 20–350 mg/L 2. 0.2–1 g/L 3. 4–10 4. 30–60 °C 5. 0–180 min 6. 100 rpm	87.50%	193.2 mg/g	Freundlich Langmuir Temkin	Pseudo-second-order Pseudo-first-order	[138]
Quaternary ammonium magnetic chitosan	Congo red	1. 50–250 mg/L 2. 0.05–0.35 g/L 3. 2–11 4. 40–60 °C 5. 10–1440 min 6. 130 rpm	99.8%	632.80 mg/g	Langmuir Freundlich	Pseudo-second-order Pseudo-first-order Intraparticle diffusion	[139]
Chitosan/dimethyl diallyl ammonium chloride/carboxymethyl cellulose	Malachite green (MG) Rhodamine B (RB) Methylene blue (MB) Bright yellow 7 GL (BY) Methyl orange (MO) Acid blue 113 (AB113) Acid black 172 (AB172) Reactive black 5 (RB5)	1. 100 mg/L 2. 0.4 g/L 3. 3–11 4. Ambient 5. 0–120 min 6. No stirring	Not available	MG 12.30 mg/g MB 16.90 mg/g RB 21.10 mg/g BY 49.20 mg/g AB172 204.80 mg/g AB113 220.00 mg/g RB5 294.70 mg/g MO 126.50 mg/g	Langmuir Temkin Freundlich	Pseudo-second-order Pseudo-first-order Intraparticle diffusion	[140]
Chitosan/diatomite/calcium alginate	Congo red	1. 20–150 mg/L 2. 4 g/L 3. 7 4. 20 °C 5. 0–24,000 min 6. No stirring	89.90%	38.84 mg/g	Langmuir Freundlich	Pseudo-second-order Pseudo-first-order	[141]
Chitosan/poly(methacrylate)	Bromocresol green	1. 5–100 mg/L 2. 2 g/L 3. 1–10 4. Ambient 5. 2–120 min 6. No stirring	99%	39.84 mg/g	Freundlich Langmuir	Pseudo-second-order Pseudo-first-order	[142]
Chitosan/MgO	Reactive blue 19	1. 100–700 mg/L 2. 1.33–10.66 g/L 3. 3–9 4. 18–38 °C 5. 30–180 min 6. 150 rpm	77.62%	512.82 mg/g	Freundlich Langmuir	Pseudo-second-order Pseudo-first-order	[143]
Chitosan/silica/Fe_3_O_4_	Methylene blue	1. 100–550 mg/L 2. 1 g/L 3. 2–9 4. 25 °C 5. 20–160 min 6. Under shaking	97%	245 mg/g	Langmuir Freundlich	Pseudo-second-order Pseudo-first-order	[144]
2-hydroxy-1-naphthaldehyde linked Fe_3_O_4_/chitosan/polyacrylamide	Everzol black	1. 10–100 mg/L 2. 0.5–2.5 g/L 3. 2–12 4. 10–35 °C 5. 10–20 min 6. No stirring	94.87%	63.69 mg/g	Langmuir Freundlich Temkin	Pseudo-second-order Pseudo-first-order	[145]
2-acrylamido-2-methylpropane sulfonic acid/acrylic acid/chitosan/ magnetite	Methylene blue	1. 100–1200 mg/L 2. 1 g/L 3. 2–11 4. 25–45 °C 5. 0–1440 min 6. 150 rpm	Not available	925.9 mg/g	Langmuir Freundlich Dubinin–Radushkevich	Pseudo-second-order Pseudo-first-order Intraparticle diffusion	[146]
Polyvinyl alcohol/ chitosan/silver nanoparticles	Congo red (CR) Crystal violet (CV)	1. 2–18 mg/L 2. 1 g/L 3. 4–9 4. 25–50 °C 5. 0–2880 min 6. 80 rpm	CR 99.91% CV 94.7%	CR 17.98 mg/g CV 11.365 mg/g	Langmuir Freundlich	Pseudo-first-order Pseudo-second-order	[147]
Oxalic acid/chitosan/alumina ceramic	Reactive red 195	1. 70–500 mg/L 2. 0.43–4.25 g/L 3. 2–12 4. 25–45 °C 5. 0–1440 min 6. 125 rpm	>80%	345.3 mg/g	Langmuir Freundlich Temkin Redlich–Peterson Dubinin–Radushkevich	Pseudo-second-order Pseudo-first-order Elovich Intraparticle diffusion Mixed surface reaction–diffusion-controlled kinetic	[148]
Chitosan/iron oxide	Methylene blue	1. 10 mg/L 2. 0.4–6 g/L 3. 1–9 4. 25 °C 5. 0–60 min 6. 300 rpm	80%	5.12 mg/g	Langmuir Freundlich Temkin Dubinin–Radushkevich	Pseudo-second-order Elovich Power function Pseudo-first-order	[149]
Chitosan/poly (acrylic acid-co-N-isopropylacrylamide)/graphite oxide	Methylene blue (MB) Fuchsin basic (FB)	1. 100–4000 mg/L 2. 0.13–1.33 g/L 3. Natural 4. 25–45 °C 5. 0–1440 min 6. No stirring	MB 77.7% FB 57.9%	MB 2748.1 mg/g FB 2246.9 mg/g	Redlich–Peterson Langmuir Freundlich	Pseudo-second-order Pseudo-first-order	[150]
Chitosan/magnetic graphene oxide	Congo red	1. 150–300 mg/L 2. 2.5–25 g/L 3. 2–11 4. 25–55 °C 5. 0–1440 min 6. 120 rpm	99.27%	395.8 mg/g	Langmuir Freundlich	Pseudo-second-order Pseudo-first-order	[151]
Chitosan/citric acid modified pistachio shell/halloysite nanotubes/glutaraldehyde	Methylene blue	1. 25–250 mg/L 2. 0.5–3.0 g/L 3. 2–8 4. 25–45 °C 5. 15–240 min 6. 150 rpm	>75%	111.14 mg/g	Langmuir Freundlich Temkin Dubinin–Radushkevich	Pseudo-second-order Pseudo-first-order	[152]
Chitosan/laponite	Methylene blue (MB) Congo red (CR)	1. 100 mg/L 2. 1 g/L 3. 2–10 4. 20–60 °C 5. 0–180 min 6. Under stirring	Not available	MB 563.6 mg/g CR 390.3 mg/g	Langmuir Freundlich	Pseudo-second-order Pseudo-first-order	[153]
Amino hydroxyapatite/sodium tripolyphosphate/chitosan	Reactive violet 5R	1. 50–700 mg/L 2. 1.25–10 g/L 3. 3–9 4. 25 °C 5. 5–600 min 6. No stirring	98%	365 mg/g	Langmuir Freundlich Temkin	Pseudo-second-order Pseudo-first-order Elovich	[154]
Chitosan/tripolyphosphate/zinc oxide	Methyl orange	1. 150–350 mg/L 2. 1–25 g/L 3. 2–12 4. 25–45 °C 5. 0–210 min 6. No stirring	99.87%	185.2 mg/g	Freundlich Langmuir	Pseudo-second-order Pseudo-first-order	[155]
Sulfonated chitosan/montmorillonite	Methylene blue	1. 40–400 mg/L 2. 0.2–3 g/L 3. 4–12 4. 30–60 °C 5. 0–180 min 6. 200 rpm	>95%	141.2 mg/g	Temkin Freundlich Langmuir	Pseudo-second-order Pseudo-first-order	[156]
Chitosan/magnetite	Congo red	1. 250 mg/L 2. 150 mg 3. Natural 4. Ambient 5. 0–120 min 6. Under stirring	Not available	22.6 mg/g	Not available	Pseudo-second-order Pseudo-first-order	[157]
Ethyl acrylate/chitosan	Methylene blue	1. 20–100 mg/L 2. 0.5–1.5 g/L 3. 7–11 4. 29.2 °C 5. 0–120 min 6. No stirring	98.4%	384.61 mg/g	Langmuir Freundlich Temkin	Not available	[158]
Chitosan/acrylic acid/acrylamide/bentonite	Malachite green (MG) Methyl violet (MV)	1. 2.5–20 mg/L MG; 200–1000 mg/L MV 2. Not available 3. 3–10 4. Ambient 5. 0–1440 min 6. Under stirring	MG 93% MV 96.5%	MG 492 mg/g MV 482 mg/g	Langmuir–Freundlich	Pseudo-second-order Pseudo-first-order	[159]
Poly(itaconic acid) and poly(acrylic acid) chitosan/magnetite	Methylene blue	1. 0.1–5 mM 2. 0.167 g/L 3. 7 4. 20 °C 5. 0–1440 min 6. Under shaking	>99%	470.2 mg/g	Sips Langmuir Freundlich	Pseudo-second-order Pseudo-first-order	[160]
Chitosan/glycidyl methacrylate/FeCl_3/_KPS	Reactive red 120 (RR) Indigo carmine (IC)	1. 5–600 mg/L 2. 0.0125–0.25 g 3. 3–9 4. 5–50 °C 5. 5–300 min 6. No stirring	RR 100% IV 100%	RR 241 mg/g IC 185 mg/g	Langmuir Freundlich Dubinin–Radushkevich	Pseudo-second-order Pseudo-first-order	[161]
Chitosan/Fe_3_O_4_	Reactive black 5 (RB5) Methyl orange (MO)	1. 25–300 mg/L 2. 3.33 g/L 3. 4–10 4. 25 °C 5. 1–180 min 6. 18 rpm	Not available	RB5 53.02 mg/g MO 70.85 mg/g	Langmuir Freundlich Redlich–Peterson Temkin	Pseudo-second-order Pseudo-first-order Intraparticle diffusion	[162]
Magnetite/amino-silica/chitosan/diethylenetriaminepentaacetic acid/graphene oxide	Basic blue 41	1. 8–600 mg/L 2. 0.12–2.48 g/L 3. 4–10 4. 17.8–68.2 °C 5. 0–60 min 6. 150 rpm	95%	55.87 mg/g	Langmuir–Freundlich Toth Extended Langmuir Temkin Redlich–Peterson Freundlich Langmuir Modified Langmuir	Pseudo-second-order Pseudo-first-order Elovich Mixed 1,2-order Fractal-like pseudo-first-order Fractal-like pseudo-second-order	[163]

* 1. Dye initial concentration; 2. sorbent dosage; 3. pH; 4. temperature; 5. contact time; 6. stirring speed.

Our investigation of the research studies included in the present review also allowed the identification of several mechanisms governing the adsorption process. The affinity between the adsorbents based on chitosan and nanoparticles and the persistent dye pollutants is often explained by electrostatic interactions, hydrogen bonding, ion exchange and dipole–dipole interactions. Jin et al. [164] report that pore filling is answerable for the adsorption of Congo red on MIL-53(Fe)/chitosan hydrogel. The porous nature of the adsorbent ensures a good diffusion of the dye and accelerate the process. Ionic interactions with negative electrostatic potential occurring between the sulfonic acid groups of Congo red and the cations of the hydrogel surface are also mentioned. Yoshida hydrogen bonding taking place between the hydrogen of the dye hydroxyl groups and π electron from the adsorbent aromatic ring affects equally the adsorption. Kafil et al. [165] used an adsorbent made of chitosan and carbon nanoflowers to eliminate Acid black 1 and Congo red dyes from aqueous media. They established that the resulting material has a positively charged surface. The anionic dyes chosen for tests possess negative charges interacting electrostatically with the adsorbent. Hydrophobic interactions of the nonpolar tail of target compounds and the adsorbent material surface and hydrogen bond caused by acting as hydrogen donor and receptor of the adsorption process participants were considered as other possible mechanisms. Kaur et al. [166], who prepared a hydrogel made of chitosan, carboxymethyl cellulose and bentonite and evaluated its ability to retain Rose Bengal and Malachite green dyes, propose electrostatic interactions, dipole–dipole interactions, ion exchange, and hydrogen bonding as leading mechanisms. Barus et al. [167] examined the adsorption capacity of a nanocomposite hydrogel prepared of chitosan, cellulose nanofiber, and graphene oxide against Methylene blue dye and emphasized that the main adsorption mechanism is represented by electrostatic interactions occurring between the adsorbent negatively charged surface and the positively charged dye molecule. Electrostatic interactions and hydrogen bonding are the main mechanisms suggested also by Șenol et al. [168], Kumar et al. [169], or Parshi et al. [170] as explanations for the high recorded removal of various dyes, such as Auramine O, Congo red, Direct blue, Crystal violet, or Rose Bengal by the prepared materials.

### 2.4. Adsorption Isotherms

Acquired at a constant pressure and temperature, the isotherms are curves that describe phenomena pertaining to a substance’s mobility, release, or retention in a solid state. The interactions between the adsorbate existing in a solution and the adsorbent with which the solution is put in contact occur for a specific period of time until an equilibrium is attained and the adsorbate concentration at the interface balances the concentration of the adsorbate in the solution. At the moment, a wide range of isotherms with two, three, or multiple parameters (Table 4) are available.

Firstly used for gass adsorption on solids [171], the Langmuir isotherm makes the assumption that there is no lateral particle contact and that the reactive groups are distributed uniformly throughout the adsorbent surface. The adsorption of a considered compound from a solution is a monolayer and takes place on a finite number of similar active sites. Oppositely, the Freundlich model [172] postulates that the adsorbent surface is heterogeneous. Therefore the adsorption is nonuniform, multilayer, and occurs on active sites with different energy values. The Temkin isotherm [173] assumes that the reduction of the adsorption heat of surface molecules is linear, the binding energies of the adsorbent surface are uniformly distributed, and that interactions happen between the adsorbate and the adsorbent. The Dubinin–Radushkevich isotherm [174] is based on the hyphothesis that the pores of an adsorbent are distributed according to Gaussian energy distribution and distinguish between chemisorption and physisorption. Toth’s model [175] is founded on the Langmuir isotherm. It describes the adsorption at extreme concentrations (elevated or reduced) and considers the adsorbent surface as heterogeneous. The Redlich–Peterson isotherm [176] is suitable for solutions containing solutes in wide ranges of concentrations. Being a combination of the Langmuir and Freundlich models, it is useful both for homogeneous and heterogeneous systems. The Sips model [177] ameliorates Freundlich’s isotherm in terms of an unceasing increase in the adsorbent amount when an augmentation of the adsorbate concentration is detected.

Most of the records included in the present review reveal that usually Langmuir and Freundlich isotherms are able to express the retention of the emergent organic pollutants on the adsorbents [178,179], but other models were tested likewise and served for understanding the process [36,37,180,181,182,183].

For instance, Gul et al. [184] report that their adsorbent represented by chitosan with black iron oxide cross-linked with graphene oxide removed Methyl violet and Alizarin yellow from aqueous solutions. A monolayer coverage specific to the Langmuir isotherm illustrated the adsorption at low initial concentrations. Up to a certain limit (24 mg/L for Methyl violet and 26 mg/L for Alizarin yellow), the adsorption capacities increased considerably with an increase in concentrations of both dyes and remained constant after that. The Freundlich isotherm fit well the experimental data only for Alizarin yellow.

The Langmuir model was the best fit equation also in the case of the removal of Methylene blue by the hydrogel composed of chitosan and titanium dioxide prepared by Zango et al. [185]. The calculated Langmuir constant values were inferior to unity, implying that the adsorption was favorable.

Jahanbakhsh et al. [186] obtained a nanocomposite by cross-linking β-cyclodextrin on chitosan by using epichlorohydrin in the presence of magnetic particles. Its ability to retain Methyl orange was better expressed by the Langmuir than by the Freundlich model.

Tran et al. [35] disclose that the adsorption of Methylene blue on chitosan—graphene oxide hydrogel is better described by the Freundlich model than by the Langmuir isotherm, suggesting a multilayer adsorption mechanism.

In the case of the study published by Sabzevari et al. [187], who synthesized an adsorbent of graphene oxide and chitosan and tested it for the removal of Methylene blue, it can be noted that the Sips isotherm was used to estimate the uptake properties of the obtained material, with the maximum adsorption capacity calculated being 408.7 mg/g.

Tamer et al. [188] tested four different isotherms, namely the Langmuir, Freundlich, Redlich–Peterson, and Sips, in order to analyze the adsorption behavior of Methylene blue on a hydrogel based on graphene oxide, chitosan, acylamide, and itaconic acid. They arrived at the conclusion that the Langmuir is the most adequate to fit the experimental data.

Ali et al. [136] conducted a research on the adsorption of Astrazon blue and Lerui acid brilliant blue dyes on amphoteric superparamagnetic nanocomposite hydrogels based on chitosan. They dedicated a part of their study to establish which isotherm can accurately describe the process. Different models, such as the Langmuir, Freundlich, Elovich, Temkin, Dubinin–Radushkevich, and Harkin–Jura, were chosen for this purpose, and the results revealed as appropriate the Langmuir equation since it was the closest one to the experimentally aquired data.

Table 3 centralizes the isotherm models reported by other researchers as describing the adsorption behavior of tested dyes on the synthesized adsorbents. The isotherms are exposed in the order of their accuracy along with the information obtained in the experiments.

### 2.5. Kinetic Studies

Knowledge of the adsorption mechanism and possible regulating phases in the process are obtained also by studying the effect of time on the elimination of dyes by the adsorbent materials. Various kinetic models are applied in the investigation of the adsorption performance (Table 5).

Among the most encountered equations, one can foremost cite pseudo-first-order and pseudo-second-order kinetics. The primary one is dedicated to the adsorption in heterogeneous systems and is related to the diffusion of the adsorbate from the initial solution to the solid adsorbent. Its validity is confirmed only at reduced concentrations of the compound to be adsorbed or at elevated concentrations of the adsorbent material. The second mentioned kinetic model assumes that the chemisorption is the adsorption-rate controlling step, and it is in agreement with monolayer or multilayer sorption or with the irreversibility of sorption–desorption cycles [189].

Alzahrani et al. [190] fabricated an adsorbent with polyaniline, chitosan, graphene oxide, and oxidized single-wall nanotubes and used it to adsorb Acid red 1 and Brilliant green dyes. They studied the time impact on the adsorption efficiency. They applied different kinetic equations and exposed pseudo-second-order as being the most appropriate.

Blanco et al. [135] manufactured an adsorbent of chitosan, choline chloride, urea, deep eutectic solvent, and iron oxide and put it in contact with solutions of Acid blue 80 dye. The conducted kinetic study revealed that both pseudo-first-order and pseudo-second-order models fitted the obtained data, while Elovich kinetic and intraparticle diffusion were not considered suitable to explain the process.

Hasan et al. [134] removed Malachite green from an aqueous solution with an adsorbent composed of polyacrylamide, chitosan, and γ-Fe_2_O_3_. In order to establish the rate-determining step, they applied four kinetic models: pseudo-first-order, pseudo-second-order, Elovich, and Weber–Morris intraparticle diffusion. They concluded that pseudo-second-order is adequate to describe the entire process in comparison with the Weber–Morris intraparticle diffusion equation that is appropriate only in the adsorption initial stage and with the Elovich model that cannot define the process.

Abootorabi et al. [133] prepared a nanocomposite using chitosan and zero-valent iron and realized adsorption experiments with Direct red 81 dye as the target pollutant. Their research surveyed the process with the help of pseudo-first-order and pseudo-second-order kinetic models. They observed that the adsorption fits well the pseudo-first-order kinetic. Moreover, they declare that the dye removal takes place in three steps: migration of the pollutant from the solution to the adsorbent surface, transfer of the dye in the adsorbent pores, and adsorption of the dye inside the material.

Myneni et al. [132] carried out an experimental program with an adsorbent made from chitosan and magnesium oxide nanoparticle and Methyl orange as the model molecule. The adsorption was analyzed with pseudo-first-order, pseudo-second-order, Elovich, and intraparticle diffusion kinetics. The results highlight that the pseudo-second-order model is followed.

Calderon et al. [148] tested the performance of oxalic acid-chitosan-alumina ceramic adsorbent to retain Reactive red 195 dye. Their kinetic exploration was conducted with several different models (pseudo-first-order, pseudo-second-order, Elovich, intraparticle diffusion, mixed surface reaction, and diffusion-controlled kinetic model) of which the pseudo-second-order model provided the most accurate fit with the experimental data.

Multiple other similar disclosments can be accessed in Table 3. The order of the available models respects the appropriateness of the kinetic equations.

### 2.6. Thermodynamic Studies

The dye adsorption process by the hydrogels prepared by combining chitosan with nanoparticles is dependent also on the working temperature (Table 3). Its variation aids to establish entropy (Δ*S*^0^), enthalpy (Δ*H*^0^), and Gibs free energy (Δ*G*^0^) changes and gives information about the endothermal or exothermal nature of the process.

Equations (1)–(3), in which *R* is the gas constant (8.314 J/mol), *T* is the temperature (K), *K_0_* is the distribution coefficient, and *E_a_* is the activation energy (kJ/mol), serve to calculate the thermodynamic parameters.

(1) $$∆G^{0}=-R\cdot T\cdot lnK_{0}$$

(2) $$lnK_{0}=\frac{∆H^{0}}{R\cdot T}+\frac{∆S^{0}}{R}$$

(3) $$E_{a}=∆H^{0}+R\cdot T$$

Many of the papers retained for exploration in this review publish results for thermodynamic studies.

Saadat et al. [145] obtained an adsorbent based on 2-hydroxy-1-naphtaldehyde-linked Fe_3_O_4_/chitosan-polyacrylamide and examined its ability to retain Everzol black. Three temperatures (283 K, 293 K, and 308 K) were used for thermodynamic analyses. They reveal positive values for the change of enthalpy and for the change of enthropy and a negative value for the change of Gibs free energy. Therefore, the adsorption process was determined as being an endothermic, disordered, and spontaneous one.

Comparable outcomes are disclosed by Nga et al. [143], who synthesised an adsorbent from chitosan and magnesium oxide and applied it in the adsorption of Reactive blue 19 dye. The range temperature chosen for thermodynamics studies was between 291 K and 311 K, with increments of 10 K.

Rastgordani and Zolghamein [127] carried out adsorption experiments with a hydrogel consisting of chitosan and hydroxyapatite and Titan yellow and Reactive blue 4 dyes as target compounds. The thermodynamic investigation (with a temperature between 298 K and 328 K) indicated that the process is endothermic and spontaneous.

Slightly different results are reported by Khushbu and Jindal [125], who conducted the preparation of an adsorbent from chitosan, graphene oxide, and bentonite, which was then used to adsorb Methylene blue and Xylenol orange dyes. According to the exhibited data, the adsorption process was classified as exothermic and spontaneous.

### 2.7. Hydrogels’ Reusability

The examination of the articles included in the actual review had among its focal points the possibility of reusing the adsorbents involved in the process of removing refractory chemicals from water by adsorption. As further detailed, in most cases, the adsorbents are washed with water, then submerged in different eluting solvents and reintroduced to the contaminated solutions after desorption. The procedure is resumed until the adsorbents are not able to eliminate the target molecules or until their degradation is observed.

Saharan et al. [129] fabricated an adsorbent from chitosan and silica, put it in contact with Food green 3 dye, and, after adsorption, they regenerated it. for this purpose, they used a sodium hydroxide solution as desorbent and dried the material at 80 °C. After four cycles of adsorption–desorption, 85% of the retained dye was removed by NaOH. Nonetheless, it must be pointed out that about 15% of the pollutant could not be desorbed.

Patel and Patel [128] obtained a composite hydrogel (with chitosan and Fe_3_O_4_) and investigated its ability to adsorb Methylene blue and Congo red dyes. The desorption in their study was realized in an acidic environment for Methylene blue and in basic media for Congo red. After ten cycles of adsorption–desorption, the adsorbent was still capable of removing more than 60% of the pollutants.

Al-Wasidi et al. [98] give information about a hydrogel made of chitosan, Fe_3_O_4,_ and graphene oxide and its utilization in the adsorption of Eriochrome black T and Methylene blue dyes. For the desorption, they used HCl for Eriochrome black T and NaOH for Methylene blue. After 24 h, the adsorbent was dried and reused. Six cycles of adsorption–desorption were possible in these conditions.

Xu et al. [96] obtained microspheres of a chitosan-based magnetic adsorbent which efficaciously adsorbed Acid green 25 and Reactive blue 19 dyes. Alkaline media assured an efficient desorption. Five cycles of adsorption–desorption were carried out with high recovery efficiencies.

As can be seen, the related data support the fact that the hydrogels based on chitosan and nanoparticles can be successfully applied in the adsorption processes of dyes from contaminated water and regenerated for several reuse cycles.

## 3. Conclusions and Perspectives

This review provides a summary of the synthesis and characterization of hydrogels obtained by using chitosan and nanoparticles as raw materials. It systemizes the influence of the main parameters implicated in the applications of these hydrogels as adsorbents for dye model molecules from water matrices. Special attention was directed to the understanding of the aspects concerning the isotherm and kinetic models as well as the thermodynamic behavior in order to assess the challenges raised by the development of an adequate adsorption process. Regeneration and reusability of the adsorbent hydrogels were also discussed. The present paper offers the necessary knowledge for creating other innovative hydrogels with the attended functionality in terms of dyes’ retention and can constitute a useful tool in overcoming the difficulties and shortfalls that may arise in practice.

The conducted investigation allowed the identification of some limitations that should be considered in future studies:

- In order to increase the adsorption capacity, it is recommended to modify the hydrogels’ structure by adding new components, such as combinations of clay and metal oxides;
- The efficacy of chitosan nanoparticles-based hydrogels in treating real industrial wastewaters that contain a variety of contaminants besides dyes should be the subject of thorough research;
- It is advised to test the ability of hydrogels to remove an entire panoply of water contaminants at the same time;
- Optimization steps of the hydrogels’ preparation and of the adsorption process by using various methods, such as response surface methodology or artificial neural network, should be compulsorily conducted;
- Further complex investigations should be carried out on the hydrogels’ regeneration and reusability.

## 4. Methodology

**Figure 6:**
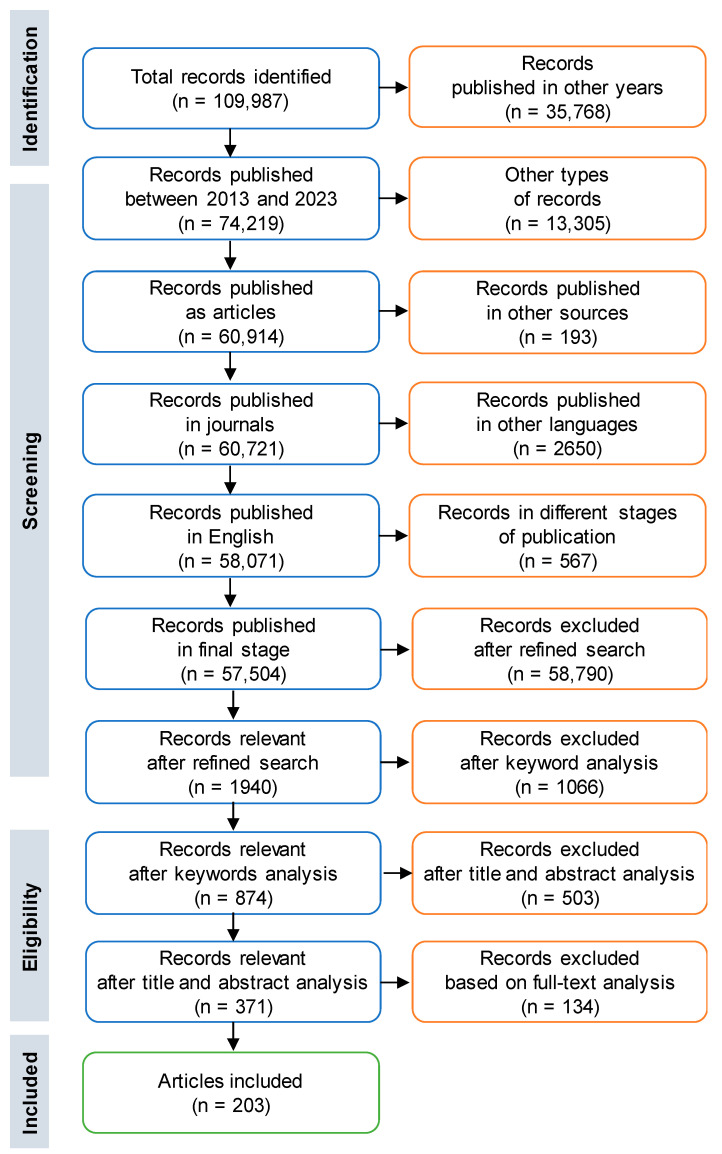
PRISMA diagram of the articles chosen for the study.

The preferred reporting items for systematic review and meta-analysis (PRISMA) [38] were followed in the design of the review to guarantee a meticulous and objective search for the pertinent literature. The steps in selecting the pertinent articles for this study are depicted in Figure 6.

We explored the literature for information on hydrogels based on chitosan and nanoparticles as well as the existence of their use for dye adsorption from water. The Scopus database was chosen for this purpose. Initially, the keyword “chitosan” was considered for the search. Only publications published between 2014 and 2023, containing primary data included in articles appearing in journals were taken into consideration. Review studies, those available in languages other than English, or those that did not have available titles, abstracts, and complete texts were excluded. After that, a refined search was conducted within the results returned by the Scopus database, with the included keywords being “nanoparticle”, “hydrogel”, “pollutant”, “dye”, and “adsorption”. A screening of the list of relevant keywords offered by Scopus was conducted to exclude unrelated papers. Other records were excluded after reading their titles, abstracts, and keywords. Afterwards, a careful analysis of the full texts was conducted and led to the exclusion of the irrelevant papers. The outcomes of the retained articles were then summarized and included in the present review.

## Figures and Tables

**Table 1:** Classification of dyes based on the nature of chromophore groups.

Dye Class	Examples	
Azo dyes	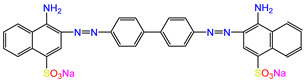	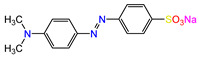
	Congo red	Methyl orange
Anthraquinone dyes	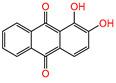	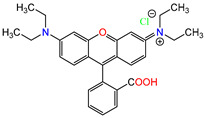
	Alizarin	Rhodamine B
Indigo dyes	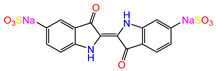	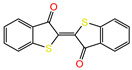
	Indigo carmine	Thioindigo
Xanthene dyes	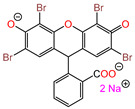	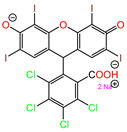
	Eosin	Rose Bengal
Phthalocyanine dyes	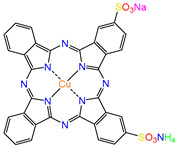	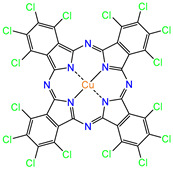
	Direct blue 199	Phthalocyanine green
Nitro dyes	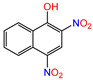	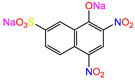
	Martius yellow	Naphtol yellow
Nitroso dyes	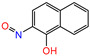	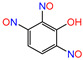
	2-nitroso-1-naphtol	Fast green O
Arylmethane dyes	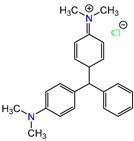	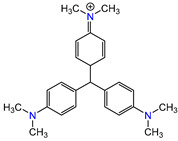
	Malachite green	Crystal violet

**Table 2:** Classification of dyes based on the nature of auxochrome groups.

Dye Class	Examples	
Acid dyes (anionic dyes)	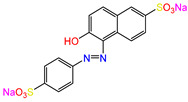	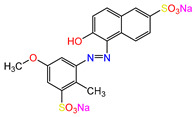
	Sunset yellow	Allura red
Basic dyes (cationic dyes)	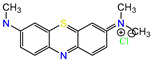	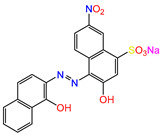
	Methylene blue	Eriochrome black T
Reactive dyes	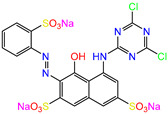	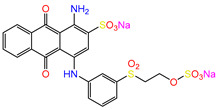
	Reactive red 1	Reactive blue 19
Direct dyes	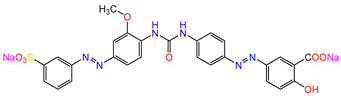	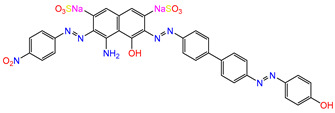
	Direct yellow 44	Direct green 6
Vat dyes	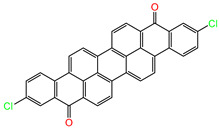	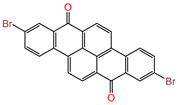
	Vat violet 1	Vat orange 1
Sulfur dyes	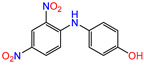	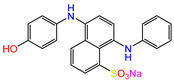
	Sulfur blue	Sulfur brilliant green
Disperse dyes	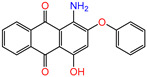	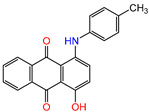
	Disperse red 60	Disperse blue 72

**Table 4:** Equations (nonlinear forms) of the equilibrium isotherms reported in the records included in the review.

Equilibrium Isotherm	Equation	Parameters’ Significance *
Two-terms isotherms		
Langmuir	$Q_{e}=\frac{Q_{mL}\cdot K_{L}\cdot C_{e}}{1+K_{L}\cdot C_{e}}$	*Q_mL_*—maximum Langmuir uptake, mg/g *K_L_*—Langmuir constant, L/mg
Extended Langmuir	$Q_{e,j}=\frac{Q_{mL,i}\cdot K_{L,i}\cdot C_{e,i}}{1+\sum_{j=1}^{N}K_{L,j}\cdot C_{e,j}}$	*Q_mL_*—maximum Langmuir uptake for component, mg/g *K_L_*—Langmuir constant for component, L/mg
Modified Langmuir	$Q_{e,j}=\frac{Q_{mL,i}\cdot K_{L,i}\cdot \left(C_{e,i}/\eta_{i}\right)}{1+\sum_{j=1}^{N}K_{L,j}\cdot \left(C_{e,j}/\eta_{j}\right)}$	*Q_mL_*—maximum Langmuir uptake for component, mg/g *K_L_*—Langmuir constant for component, L/mg *η*—additional interaction factor
Freundlich	$Q_{e}=K_{F}\cdot C_{e}^{1/n_{F}}$	*K_F_*—Freundlich constant, (mg/g)(L/mg)^1/*n*^ *n_F_*—Freundlich constant, dimensionless
Extended Freundlich	Qe,i=KF,i·Ce,i1ni+xiCe,ixi+yi·Ce,izi Qe,j=KF,j·Ce,j1nj+xjCe,jxj+yj·Ce,jzj	*K_F_*—Freundlich constant for component, L/mg *n* —Adsorption intensity for components in a solution *x_i_*, *x_j_*—Experimental constant values of plot *Q_e,i_* vs. *C_e,i_*, dimensionless *y_i_*, *y_j_*—Experimental constant values of plot *Q_e,j_* vs. *C_e,j_*, dimensionless *z_i_*, *z_j_*—Experimental constant values of plot *Q_e,i_* vs. *C_e,i_*, dimensionless
Langmuir–Freundlich	$Q_{e}=\frac{Q_{mLF}\cdot {\left(K_{LF}\cdot C_{e}\right)}^{n}}{1+{\left(K_{LF}\cdot C_{e}\right)}^{n}}$	*Q_mLF_*—maximum Langmuir–Freundlich uptake, mg/g *K_LF_*—Langmuir–Freundlich constant, L/g *n*—heterogeneity index, dimensionless
Modified Langmuir–Freundlich	$Q_{e}=\frac{Q_{mLF}\cdot {\left(C_{e}\cdot k_{a}\right)}^{n}}{1+{\left(C_{e}\cdot k_{a}\right)}^{n}}$	*Q_mLF_*—maximum Langmuir–Freundlich uptake, mg/g *k_a_*—Affinity coefficient for adsorption, L/mg
Temkin	$Q_{e}=\frac{R\cdot T}{b_{T}}\cdot ln(K_{T}\cdot C_{e})$	*R*—gas constant, *R* = 8.314 J/(mol K) *T*—temperature, K *K_T_*—Temkin constant, L/mg *b_T_*—Temkin constant, J/mg
Hill	$Q_{e}=\frac{Q_{S_{H}}{\cdot C}_{e}^{n_{H}}}{K_{D}+C_{e}^{n_{H}}}$	*Q_SH_*—Hill maximum uptake saturation, mg/L *K_d_*—Hill constant *n_H_*—Hill cooperativity coefficient of the binding interaction
Flory— Huggins	$Q_{e}=Q_{mFH}^{\left(1-n\right)}\cdot {\left(Q_{mFH}-Q_{e}\right)}^{n}\cdot K_{FH}\cdot C_{e}$	*Q_mFH_*—Flory–Huggins maximum adsorption capacity, mg/g *K_FH_*—Flory–Huggins equilibrium constant, L/mg *n*—exponent, dimensionless
Dubinin– Radushkevich	$Q_{e}=\left(Q_{s}\right)\exp\left(-k_{ad}\varepsilon^{2}\right)$	*Qs*—Theoretical monolayer saturation capacity, mg/g *B*—Constant of the sorption energy, mol^2^/kJ^2^ *ε*—Polanyi potential, dimensionless
Elovich	$\frac{Q_{e}}{Q_{mE}}=K_{E}\cdot C_{e}\cdot exp\left(-\frac{Q_{e}}{Q_{mE}}\right)$	*Q_mE_*—Elovich maximum adsorption capacity, mg/g *K_FG_*—Elovich equilibrium constant, L/mg
Liu	$Q_{e}=\frac{Q_{mLi}\cdot {\left(K_{g}\cdot C_{e}\right)}^{nL}}{1+{\left(K_{g}\cdot C_{e}\right)}^{nL}}$	*Q_mLi_*—Liu maximum uptake, mg/g *K_g_*—Liu constant, L/g *n_L_*—heterogeneity index, dimensionless
Fowler– Guggenheim	$C_{e}=\frac{1}{K_{FG}}\frac{Q_{e}}{Q_{FG}-Q_{e}}+exp\left(\frac{2\cdot \omega }{R\cdot T}\cdot \frac{Q_{e}}{Q_{FG}}\right)$	*Q_FG_*—Fowler–Guggenheim maximum adsorption capacity, mg/g *K_FG_*—Fowler–Guggenheim equilibrium constant, L/mg *R*—gas constant, *R* = 8.314 J/(mol K) *T*—temperature (K) *ω*—interaction energy between adsorbed molecules, Kj/mol
Harkin–Jura	$\frac{1}{Q_{e}^{2}}=\frac{B}{A}-\left(\frac{1}{A}\right)\cdot logC_{e}$	*A*—Harkin–Jura constant, dimensionless *B*—Harkin–Jura constant, dimensionless
Three-terms isotherms		
Toth	$Q_{e}=\frac{Q_{To}\cdot C_{e}}{{\left(\frac{1}{K_{To}}+{C_{e}}^{n_{To}}\right)}^{1/n_{To}}}$	*Q_To_*—Toth maximum uptake, mg/g *K_To_*—Toth constant, L/mg *n_To_*—Toth constant, dimensionless
Sips	$Q_{d,t}=\frac{Q_{S}\cdot {\left(K_{S}\cdot C_{e}\right)}^{n_{S}}}{1+{\left(K_{S}\cdot C_{e}\right)}^{n_{S}}}$	*Q_S_*—Sips maximum uptake, mg/g *K_S_*—Sips constant, L/mg *n_S_*—Sips constant, dimensionless
Redlich– Peterson	$Q_{e}=\frac{K_{R}{\cdot C}_{e}}{1+a_{R}{\cdot C}_{e}^{b_{g}}}$	*K_R_*—Redlich–Peterson constant, L/g *a*_R_—Redlich–Peterson constant, 1/mg *bg*—Redlich–Peterson exponent, dimensionless
Radke–Prausnitz	$Q_{e}=\frac{Q_{MRP}\cdot K_{RP}\cdot C_{e}}{{\left(1+K_{RP}\cdot C_{e}\right)}^{MRP}}$	*Q_MRP_*—Radke–Prausnitz maximum adsorption capacity, mg/g *K_RP_*—Radke–Prausnitz equilibrium constant, dimensionless *MRP*—Radke–Prausnitz exponent, dimensionless

* Common symbols’ significance: *Q_e_*—adsorbate concentration in solid phase at equilibrium, mg/g; *C_e_*—adsorbate concentration in fluid phase at equilibrium, mg/L; *i*, *j* – components.

**Table 5:** Equations (nonlinear forms) of the kinetic models reported in the records included in the review.

Kinetic Model	Equation	Parameters Significance *
Pseudo-1st-order	$Q_{t}=Q_{e}\cdot (1-e^{-k_{1}\cdot t})$	*k*_1_—pseudo-first-order constant rate, min^−1^
Pseudo-2nd-order	$Q_{t}=\frac{k_{2}\cdot Q_{e}^{2}\cdot t}{\left(1+k_{2}\cdot Q_{e}\cdot t\right)}$	*k*_2_—pseudo-second-order constant rate, g/(mg·min)
Mixed 1,2-order	$Q_{t}=Q_{e}\frac{1-exp(-k_{1}\cdot t)}{1-f_{2}exp(-k_{1}\cdot t)}$ $f_{2}=\frac{k_{1}\cdot Q_{e}}{k_{1}+k_{2}\cdot Q_{e}}$	*k*_1_—pseudo-first-order constant rate, min^−1^ *k*_2_—pseudo-second-order constant rate, g/(mg·min)
Pseudo-*n*th-order	$Q_{t}=Q_{e}\left[1-\frac{1}{\left(1+{\left(n-1\right)}_{Q_{e}^{n-1}}\cdot k_{n}\cdot t^{1/\left(n-1\right)}\right)}\right]$	*k_n_*—pseudo-*n*th-order constant rate
Fractal-like pseudo-1st-order	$Q_{t}=Q_{e}\left[1-exp\left(\frac{-k_{1}^{\prime }}{1-h}\right)\cdot t^{\left(1-h\right)}\right]$	*k*′_1_—fractal-like pseudo-first-order constant rate, min^−(1-h)^ *h*—fractal exponent, 0 ≤ *h* ≤ 1, dimensionless
Fractal-like pseudo-2nd-order	$Q_{t}=\frac{\frac{k_{2}^{\prime }}{1-h}\cdot Q_{e}^{2}\cdot t^{\left(1-h\right)}}{1+\frac{k_{2}^{\prime }}{1-h}\cdot Q_{e}\cdot t^{\left(1-h\right)}}$	*k*_2_—fractal-like pseudo-second-order constant rate, g mg^−1^ min^−(1-h)^)
Fractal-like mixed 1,2 order	$Q_{t}=Q_{e}\left[\frac{1-exp\left(\frac{-k_{1}^{\prime }}{1-h}\right)\cdot t^{\left(1-h\right)}}{1-f_{2}\cdot exp\left(\frac{-k_{1}^{\prime }}{1-h}\right)\cdot t^{\left(1-h\right)}}\right]$	*K*′_1_—fractal-like pseudo-first-order constant rate, min^−(1-h)^ *h*—fractal exponent, 0 ≤ *h* ≤ 1, dimensionless
Intraparticle diffusion	$Q_{t}=k_{p}\cdot t^{0.5}+c$	*k_p_*—rate constant of the intraparticle diffusion kinetic model, mg/g min^1/2^ *c*—constant, dimensionless
Elovich	$Q_{t}=\frac{1}{\beta }\cdot ln(\alpha \cdot \beta \cdot t+1)$	*β*—extent of surface coverage and activation energy for chemisorption, g/mg *α*—initial adsorption rate, mg/(g·min)
Mixed surface reaction—diffusion controlled kinetic	$Q_{t}=\frac{exp(a\cdot t+b\cdot t^{0.5})}{\left(w_{eq}\cdot \exp\left(a\cdot t+b\cdot t^{0.5}\right)-1\right)}$ $w_{eq}=1-\left(\frac{C_{e}}{C_{0}}\right)$ $a=k\cdot C_{0}\cdot \left(w_{eq}-1\right)$ $b=2\cdot k\cdot C_{0}\cdot t^{0.5}\left(w_{eq}-1\right)$	*C*_0_—initial concentration solute, mg/L *a*—model coefficient, mg/L *b*—model coefficient, 1/min *w_eq_*—relative equilibrium uptake
Liquid film diffusion	$\ln\left(1-\frac{Q_{t}}{Q_{e}}\right)=-k_{FD}\cdot t+C$	*K_FD_*—liquid film diffusion constant

* Common symbols’ significance: *Q_t_*—concentration in the solid phase at time *t*, mg/g; *Q_e_*—adsorbent capacity at equilibrium, mg/g; *t*—contact time, min.

## Data Availability

Not applicable.
